# A life course examination of the physical environmental determinants of physical activity behaviour: A “Determinants of Diet and Physical Activity” (DEDIPAC) umbrella systematic literature review

**DOI:** 10.1371/journal.pone.0182083

**Published:** 2017-08-07

**Authors:** Angela Carlin, Camille Perchoux, Anna Puggina, Katina Aleksovska, Christoph Buck, Con Burns, Greet Cardon, Simon Chantal, Donatella Ciarapica, Giancarlo Condello, Tara Coppinger, Cristina Cortis, Sara D’Haese, Marieke De Craemer, Andrea Di Blasio, Sylvia Hansen, Licia Iacoviello, Johann Issartel, Pascal Izzicupo, Lina Jaeschke, Martina Kanning, Aileen Kennedy, Jeroen Lakerveld, Fiona Chun Man Ling, Agnes Luzak, Giorgio Napolitano, Julie-Anne Nazare, Tobias Pischon, Angela Polito, Alessandra Sannella, Holger Schulz, Rhoda Sohun, Astrid Steinbrecher, Wolfgang Schlicht, Walter Ricciardi, Ciaran MacDonncha, Laura Capranica, Stefania Boccia

**Affiliations:** 1 Department of Physical Education and Sport Sciences, University of Limerick, Limerick, Ireland; 2 Centre de Recherche en Nutrition Humaine Rhône-Alpes, CarMeN INSERM U1060, University of Lyon1, Lyon, France; 3 Luxembourg Institute of Socio-Economic Research, Esch/Alzette, Luxembourg; 4 Section of Hygiene - Institute of Public Health, Università Cattolica del Sacro Cuore, Rome, Italy; 5 Leibniz Institute for Prevention Research and Epidemiology - BIPS, Bremen, Germany; 6 Department of Sport, Leisure and Childhood Studies, Cork Institute of Technology, Cork, Munster, Ireland; 7 Department of Movement and Sports Sciences, Ghent University, Ghent, Belgium; 8 Council for Agricultural Research and Economics -Research Centre for Food and Nutrition, Rome, Italy; 9 Department of Movement, Human and Health Sciences, University of Rome Foro Italico, Rome, Italy; 10 Department of Human Sciences, Society, and Health, University of Cassino and Lazio Meridionale, Cassino, Italy; 11 Department of Medicine and Aging Sciences, 'G. d'Annunzio' University of Chieti-Pescara, Chieta, Italy; 12 Department for Sport and Exercise Sciences, University of Stuttgart, Stuttgart, Germany; 13 Department of Epidemiology and Prevention, IRCCS Istituto Neurologico Mediterraneo: NEUROMED. Pozzilli. Italy; 14 School of Health and Human Performance, Multisensory Motor Learning Lab, Dublin City University, Dublin, Ireland; 15 Molecular Epidemiology Group, Max Delbruck Center for Molecular Medicine in the Helmholtz Association (MDC), Berlin, Germany; 16 Department for Sport Sciences, University of Konstanz, Konstanz, Germany; 17 Centre for Preventive Medicine, School of Health and Human Performance, Dublin City University, Dublin, Ireland; 18 Department of Epidemiology and Biostatics, EMGO Institute for Health and Care Research, VU University medical center, Amsterdam, The Netherlands; 19 Institute of Sport, Exercise & Active Living, Victoria University, Melbourne, Australia; 20 Department of Psychology, Bournemouth University, Bournemouth, United Kingdom; 21 Institute of Epidemiology I, Helmholtz Zentrum München, German Research Center for Environmental Health, Neuherberg, Germany; 22 Charité Universitätsmedizin Berlin, Berlin, Germany; 23 DZHK (German Centre for Cardiovascular Research), partner site Berlin, Berlin, Germany; 24 Italian National Institute of Health, Rome, Italy (Istituto Superiore di Sanita - ISS); 25 Health Research Institute (HRI), University of Limerick, Limerick, Ireland; 26 Ichan School of Medicine at Mount Sinai, New York, United States of America; Vanderbilt University, UNITED STATES

## Abstract

**Background:**

Participation in regular physical activity is associated with a multitude of health benefits across the life course. However, many people fail to meet PA recommendations. Despite a plethora of studies, the evidence regarding the environmental (physical) determinants of physical activity remains inconclusive.

**Objective:**

To identify the physical environmental determinants that influence PA across the life course.

**Methods:**

An online systematic literature search was conducted using MEDLINE, ISI Web of Science, Scopus and SPORTDiscus. The search was limited to studies published in English (January 2004 to April 2016). Only systematic literature reviews (SLRs) and meta-analyses (MAs) of observational studies, that investigated the association between physical determinants and physical activity outcomes, were eligible for inclusion. The extracted data were assessed on the importance of determinants, strength of evidence and methodological quality.

**Results:**

The literature search identified 28 SLRs and 3 MAs on 67 physical environmental characteristics potentially related to physical activity that were eligible for inclusion. Among preschool children, a positive association was reported between availability of *backyard space* and *outdoor toys/equipment* in the home and overall physical activity. The *availability of physical activity programs and equipment* within schools, and neighbourhood features such as *pedestrian and cyclist safety structure* were positively associated with physical activity in children and adolescents. Negative *street characteristics*, for example, *lack of sidewalks and streetlights*, were negatively associated with physical activity in adults. Inconsistent associations were reported for the majority of reviewed determinants in adults.

**Conclusion:**

This umbrella SLR provided a comprehensive overview of the physical environment determinants of physical activity across the life course and has highlighted, particularly amongst youth, a number of key determinants that may be associated with overall physical activity. Given the limited evidence drawn mostly from cross-sectional studies, longitudinal studies are needed to further explore these associations.

**Registration:**

PROSPERO CRD42015010616

## Introduction

Participation in regular physical activity (PA) is associated with a multitude of health benefits across the life course [1,2] and plays a key role in the prevention and management of non-communicable diseases (NCDs); including cardiovascular disease, type 2 diabetes, depression, osteoporosis and some cancers [2,3]. The World Health Organization recommends that children and adolescents (5–17 years) take part in at least 60 minutes of moderate-to-vigorous PA (MVPA) each day, while adults should engage in at least 150 minutes per week [4]. A large proportions of the population fail to meet these guidelines, with approximately one third of adults (31%) and the majority of young people aged 13–15 years (80%) worldwide classed as physically inactive [5]. Recent report cards have also indicated high levels of inactivity amongst children globally [6]. Physical inactivity also has significant economic implications. Conservative estimates indicate that physical inactivity cost health-care systems $53.8 billion globally in 2013 [7].

Researchers have identified multiple determinants of PA, ranging from proximal to distal influences within the frame of the socio-ecological model (policy, environment, inter-individual, intra-individual [8], with extensive interest in socio-demographic factors. Beside the individual-level determinants, the physical environment which individuals live in and interact with has gradually come in the forefront of PA research as a driver of physical (in)activity. Indeed, increasing use of geographical information systems, improvements in environmental exposure measurements, and developments of spatial analytic methods dedicated to evaluate the influence of environmental attributes on health (i.e. multilevel analysis, spatial autocorrelation analysis, weighted geographical models) have contributed to a dramatic increase in publications over the last years [9]. The physical environment encompasses both the natural and built environmental characteristics, as well as less tangible factors such as traffic or crime safety [10] which represent barriers and opportunities that may directly influence PA. As such, engagement in PA, including the type, frequency, intensity, and duration, has been linked to a wide range of physical environmental characteristics including the degree of urbanization of the place of residence, urban form (land use mix, street connectivity, street light), transportation network, PA equipment and natural environmental characteristics (e.g. green spaces, presence of waterway, weather) [11–13].

As a result, public health actors have advocated in favour of urban planning interventions and implementation of local solutions to promote active-friendly environments, reflecting their acknowledgement that physical environment attributes are potential levers for increasing PA at population level. However, such health promotion interventions require a comprehensive understanding of the physical attributes more conducive to PA, with the need for public health actors to reflect critically and guide the development of appropriate interventions. While multiple literature reviews have been published on the link between environment and PA, current evidence remains fragmented by the focuson specific age-categories (for example, children), PA outcomes investigated (for example, overall PA, daily steps or MVPA) or specific attributes of the physical environment, for example, reviews focused only one aspect of the physical environment individuals are exposed to, such as the educational [14] or neighbourhood setting [15]. Currently, a comprehensive evaluation of the physical environmental characteristics enhancing or reducing the practice of PA through a life course perspective is still lacking.

Recently, the European Commission endorsed a Joint Programming Initiative to increase research capacity across Member States to engage in a common research agenda on healthy diet and healthy lifestyles [16]. As a result, the **DE**terminants of **DI**et and Physical **AC**tivity **K**nowledge **H**ub (DEDIPAC-KH) project was established [17]. The current umbrella review is part of seven systematic literature reviews (SLRs) (on biological, psychological, behavioral, physical, socio-cultural, economic, policy determinants), aiming at reviewing and updating the current evidence base on the determinants of PA across the life course. The aim of this SLR umbrella is to give a comprehensive overview of any physical environmental determinant influencing PA across the life course by systematically reviewing the available evidence from previous SLRs and meta-analyses (MAs) (uniformly referred to as “reviews” in the text).

## Methods

The common protocol for the DEDIPAC umbrella systematic reviews is registered on PROSPERO (Record ID: *CRD42015010616*), the international prospective register of systematic reviews [18]. The manuscript was drafted following the Preferred Reporting Items for Systematic Reviews and Meta-Analyses (PRISMA) checklist [19].

### Search strategy and eligibility criteria

An online systematic literature search was conducted using MEDLINE, ISI Web of Science, Scopus and SPORTDiscus electronic databases to identify SLRs and MAs investigating the determinants of PA across the life course. The search was limited to studies published in the English language, during the period from January 2004 to April 2016. The search strategy was developed in MEDLINE and used as a template for the search strategies in the other databases (Table A in S1 Table). In addition to the database search, a snowball method was applied to the references of the included reviews to identify any further potentially relevant SLRs or MAs.

SLRs and MAs of observational studies that reported PA, exercise or sport as the main outcome and that reported any association between any variable potentially influencing the main outcome, across all stages of the life course, were eligible for inclusion. The following were excluded: i) SLRs and MAs of intervention studies, ii) SLRs and MAs that focused on specific population groups (e.g. patients or athletes) and iii) umbrella systematic reviews.

### Selection process

Relevant records were independently assessed by two reviewers belonging to the DEDIPAC-KH, who screened titles, and where appropriate, abstracts and full texts. Before the final inclusion or exclusion, a common decision was reached for each record. Any uncertainty or disagreement was resolved by consulting three additional authors (SB, LC, AP). Given the specific focus of the present umbrella SLR, reviews that focused on non-physical determinants of PA were not considered.

### Data extraction

For each included review, data were extracted using predefined extraction forms, developed by two authors (KA, AP) and verified by the DEDIPAC-KH. The following information was included: year of publication, type of review (SLR/MA), number of eligible primary studies included in this umbrella review over the total number of studies included in the review, continent/s of the included studies, primary study design, overall sample size, age range/mean age, gender proportion, year of publication range of included studies; outcome details, type of determinant/s, aim of the review; overall results (qualitative or quantitative), overall recommendations and limitations as provided by the review itself.

### Evaluation of importance of determinants and strength of the evidence

The importance of the determinant reported by a particular review and the strength of evidence were summarised by combining two slightly modified grading scales, used by the World Cancer Research Fund [20], and Sleddens et al. [21]. According to Sleddens [21], the codes + and ++ were used if there is an association (no matter of positive or negative). This was modified for the present review to report both the association and the direction of the association.

The importance was scored a (—) if all reviews, without exception, found a negative association between the determinant and the outcome. A (-) score was given if the negative association was found in ≥ 75% of the included reviews or of the original primary studies. The importance of the determinant was scored a (0) if the results were mixed, or more specifically, if the variable was found to be a determinant and/or reported an association (either positive or negative) in less than 75% of available reviews or of the primary studies of these reviews. The importance of a determinant was scored as (00) if all reviews, without exception, reported a null association. The importance of the determinant scored a (+) if a positive association was found in ≥75% of the reviews or of the included primary studies and a (++), if a positive association was found in all reviews, without exception.

The strength of the evidence was also summarized using the criteria adopted by Sleddens et al [21]. The strength of evidence was described as “convincing” (Ce) if it was based on a substantial number of longitudinal observational studies, with sufficient size and duration, and showing consistent associations between the determinant and PA. The strength of the evidence was defined as “probable” (Pe) if it was based on at least two cohort studies or five cross-control studies showing fairly consistent associations between the determinant and PA. The strength of the evidence was given as “limited, suggestive evidence” (Ls) if it was based mainly on findings from cross-sectional studies showing fairly consistent associations between the determinant and PA, and as “limited, non- conclusive evidence” (Lnc) if study findings were suggestive, but insufficient to provide an association between the determinant and PA.

### Quality assessment

The methodological quality of included reviews was assessed using a modified version of the AMSTAR Checklist [22]. A consensus between the DEDIPAC-KH partners was reached to modify the question referring to the presence of any conflict of interest (criteria number 11), so that the conflict of interest was evaluated for the included SLRs, and not within each individual primary study included within the reviews. Two reviewers belonging to the DEDIPAC-KH independently evaluated the included reviews. Any uncertainty and disagreement was resolved by consulting three additional authors (SB, LC, AP). The eleven criteria were evaluated and scored as 1 when the criterion was fulfilled by the analysed review or as 0 when the criterion was not applicable to or could not be answered based on the information given by the analysed review. Consequently, the total quality score for each included review ranged from 0 to 11, with the quality of the review labelled as either weak (0–3), moderate (4–7), or strong (8–11).

## Results

### SLRs and MAs selection process

**Fig 1:**
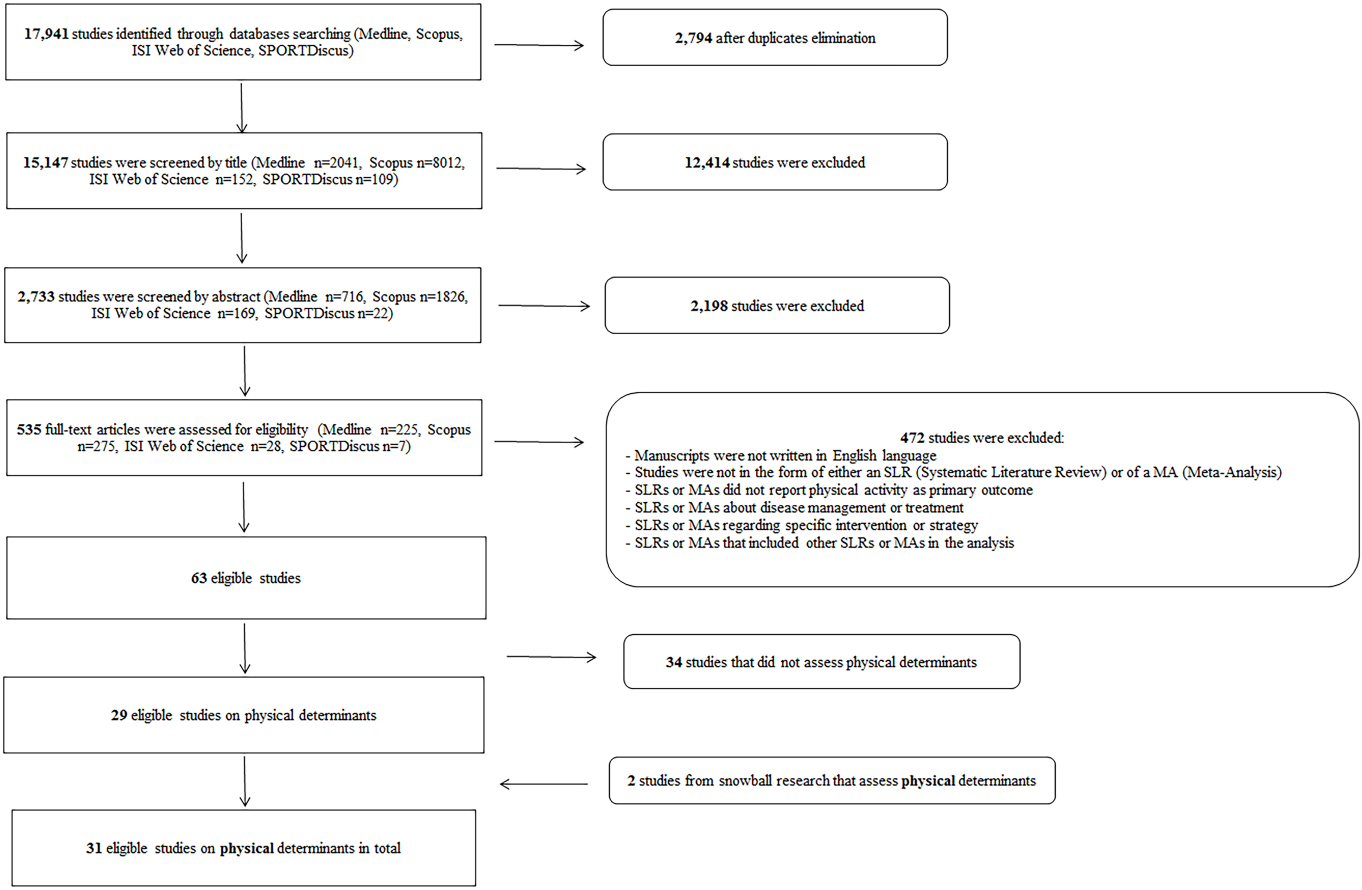
PRISMA flowchart of the literature research by database. SLR, Systematic Literature Review; MA, Meta Analysis.

Across all databases, the electronic search identified a total of 17,941 records that were potentially relevant for inclusion in the seven DEDIPAC SLRs. After the removal of duplicates, 15,147 records remained for screening. As summarised in Fig 1, 14,612 records were excluded after title and abstract screening. A total of 535 full-text records were then assessed for eligibility. The final number of SLRs and MAs eligible for the seven DEDIPAC SLRs was 63. Of these, 36 did not concern physical determinants of PA therefore 29 SLRs/MAs were included. A further two studies were identified as eligible from a snowball search of references. Therefore, 31 SLRs and MAs were included in the present umbrella SLR on physical determinants of PA.

### Characteristics of the SLRs and MAs included

The characteristics of the 31 included SLRs and MAs (28 and 3, respectively) comprising a total of 755 eligible primary studies are summarized in Table 1. Given that some of the included reviews included primary studies that examined the associations between non-physical determinants and PA, only the primary studies that included physical determinants were appraised within this umbrella review.

**Table 1:** Characteristics of the included studies (n = 31).

Author, Date (Type of review)	Number of individual studies included in the umbrella review*/total number of studies included in the review	Continent/s	Study design	Sum of the size of the individual samples included	Age range or mean (years)	Female gender %	Year publication (range)
**Babacus WS, 2012 (SLR)**[25]	7/38	Europe (n = 7)	Qualitative (n = 7)	420* (15–137)	16–90+	45–100	1980–2012
**Beets MW, 2010 (SLR)** [50]	11/80	North America (n = 11)	Cross sectional (n = 11)	6,150* (52–2,114)	8–18	N.R.	1970–2008
**Casagrande SS, 2009 (SLR)** [26]	7/10	North America (n = 7)	Cross sectional (n = 7)	5,447 (234–2,119)	18–96	56–100	2000–2005
**Coble JD, 2006 (SLR)**[27]	3/35	North America (n = 3)	Cross sectional (n = 3)	1,037 (34–653)	≥ 18	53–100	1990–2005
**Craggs C, 2011 (SLR)**[43]	8/46	North America (n = 4) Europe (n = 2) Australia (n = 2)	Longitudinal (n = 8)	11,627 (170–8,817)	≤ 9 (n = 1) 10–13 (n = 5) ≥ 14 (n = 2)	49–100	1998–2010
**Davison KK, 2006 (SLR)** [44]	32/33	North America (n = 25) Europe (n = 2) Australia (n = 5)	Longitudinal (n = 2) Cross sectional (n = 30)	44,747 (52–17,766)	3–18	N.R.	1990–2006
**De Craemer M, 2012 (SLR)**[47]	16 /43	North America (n = 5) Europe (n = 3) Australia (n = 8)	Longitudinal (n = 2) Cross sectional (n = 14)	7,238 (76–2,700)	4–6	44–55 *	1990–2010
**Ding D, 2011 (SLR)** [13]	103 /103	North America (n = 73) Europe (n = 18) Australia (n = 11) Asia (n = 1)	Longitudinal (n = 4) Cross sectional (n = 99)	(52—≥ 5,000)	3–12 (n = 56) 13–18 (n = 38) Both (n = 9)	N.R.	1993–2010
**Durand CP, 2011 (SLR)** [34]	41/44	North America (n = 28) Europe (n = 5) Australia (n = 8)	Longitudinal (n = 5) Cross sectional (n = 36)	100,622 (32–19,437)	< 18 (n = 3) 18 + (n = 31) Both (n = 7)	47–100 *	2000–2009
**D'Haese S, 2015 (SLR)** [46]	65/65	North America (n = 35) Europe (n = 17) Australia (n = 11) Asia (n = 2)	Longitudinal (n = 4) Cross sectional (n = 61)	103,086 (29–14,553)	6–12	N.R.	2000–2014
**Ferreira I, 2007 (SLR)** [41]	56/150	North America (n = 41) Europe (n = 10) Australia (n = 5)	Longitudinal (n = 5) Cross sectional (n = 51)	N.R.	3–12 (n = 31) 13–18 (n = 25)	N.R.	1980–2004
**Gustafson SL, 2006 (SLR)** [42]	3/34	North America (n = 3)	Cross sectional (n = 3)	1,551	9–12	49–51	1992–2003
**Hajna S, 2015 (MA)** [15]	6/6	Europe (n = 4) Asia (n = 2)	Cross sectional (n = 6)	1,828 (70–1,100)	18–80	41–79 *	2009–2014
**Hinkley T, 2008 (SLR)**[48]	12/24	North America (n = 10) Europe (n = 2)	Longitudinal (n = 1) Cross sectional (n = 11)	5,732 (39–3,141)	2–5	43–53	1980–2007
**Koeneman MA, 2011 (SLR)** [32]	3/30	North America (n = 2) Asia (n = 1)	Longitudinal (n = 3)	820 (95–422)	55 +	0–64	1992–2010
**Lachowycz K, 2011 (SLR)** [33]	50/60	North America (n = 30) Europe (n = 12) Australia (n = 8)	Cross-sectional (n = 50)	130,346	5–74+	0–100	2000–2010
**Larouche R, 2014 (SLR)** [40]	45/68	North America (n = 7) Europe (n = 25) Australia (n = 9) Asia (n = 3) South America (n = 1)	Cross-sectional (n = 40) Longitudinal (n = 5)	69,559 (114–7,023)	5–18	42–100	2003–2012
**Lee MC, 2008 (SLR)** [37]	25/32	North America (n = 9) Europe (n = 10) Australia (n = 5) Asia (n = 1)	Cross sectional (n = 25)	34,850 (88–10,771)	5–18	42–100 *	2002–2007
**Maitland C, 2013 (SLR)** [38]	21/49	North America (n = 6) Europe (n = 5) Australia (n = 7) Asia (n = 3)	Longitudinal (n = 3) Cross sectional (n = 18)	45,978	8–14	30–81 *	2005–2011
**McCormack GR, 2011 (SLR)** [31]	20/33	North America (n = 19) Europe (n = 1)	Cross sectional (n = 20)	56,580	18 +	27–64	1996–2010
**McGrath LF, 2015 (MA)** [39]	23/23	North America (n = 13) Europe (n = 6) Australia (n = 4)	Cross sectional (n = 23)	6,174	8–17	0–100	2005–2013
**Olsen JM, 2013 (SLR)** [29]	11 / 21	North America (n = 11)	Cross sectional (n = 4) Qualitative (n = 5) Mixed methods (n = 2)	5,847	19—(≥65)	100	2000–2010
**Pugliese J, 2007 (MA)** [45]	5 /30	North America (n = 4) Europe (n = 1)	Longitudinal (n = 1) Cross sectional (n = 4)	23,310 (21–8,834)	2–18	50–58	1960–2005
**Rich C, 2012 (SLR)** [36]	16/16	North America (n = 3) Europe (n = 12) Australia (n = 1)	Longitudinal (n = 4) Cross sectional (n = 12)	14,747 (64–5,595)	2–18	43–78 *	2002–2010
**Ridgers ND, 2012 (SLR)** [14]	16 /53	North America (n = 3) Europe (n = 9) Australia (n = 4)	Cross sectional (n = 16)	62,829 (34–36,995)	5–18	39–61	1990–2011
**Siddiqi Z, 2011 (SLR)** [23]	17/29	North America (n = 17)	Cross sectional (n = 17)	796 (16–89)	18–89	45–100	1995–2009
**Stanley AM, 2012 (SLR)** [35]	13 /22	North America (n = 5) Europe (n = 5) Australia (n = 3)	Cross sectional (n = 12 Validation study (n = 1)	37,999	8–14	48–100	1990–2011
**Tzormpatzakis N, 2007 (SLR)** [30]	5/36	Europe (n = 5)	Cross sectional (n = 5)	14,476	18–89	47–62	1993–2006
**Van der Horst K, 2007 (SLR)** [49]	9/57	North America (n = 8) Europe (n = 1)	Cross sectional (n = 9)	20,784	4–12 (n = 4) 13–18 (n = 5)	48–100 *	1999–2005
**Van Holle V, 2012 (SLR)** [24]	70/70	Europe (n = 70)	Longitudinal (n = 1) Cross sectional (n = 69)	8,367,768	18–65	36–66	2000–2011
**Wendel-Vos W, 2007 (SLR)** [28]	36/47	North America (n = 23) Europe (n = 2) Australia (n = 11)	Longitudinal (n = 1) Cross sectional (n = 35)	308,325 (107–206,992)	18+	N.R.	1980–2004

NR, Not Reported,

* where data was available—not all primary studies included in total

In most reviews the eligible primary studies came from several continents. The majority was conducted in North-America (53.6%) and Europe (31%), less from Australia/Oceania (13.54%), and little from Asia (1.7%) and South America (0.1%). The majority of included reviews (n = 26) reported findings from primary studies that were cross-sectional/longitudinal or cross-sectional only in design (n = 13 and n = 13 reviews, respectively). Where reported, the total sample size of included primary studies within reviews ranged from n = 796 [23] to n = 8,367,768 [24].

Eighteen reviews referred to primary studies that included young people only. The majority of reviews in adults (aged ≥ 18 years) included both adults and older adults [15, 25–29] yet did not provide separate analysis based on age. Two reviews focused on adult only populations (<65 years) [23,30], while it was not possible to determine the upper age limit within one review [31] One review compared adults (<50 years) with older adults (>50 years) [23], while one review focused on older adults (defined within the review as > 55 years) [32]. Two reviews included both adults and young people [33,34].

### Measurements of PA

Among the 755 primary studies included in the umbrella SLR, 119 studies from 16 reviews used objective measurements of PA (e.g. accelerometers, pedometers) [13,15,24,31,33–44]. Subjective measurements of PA (e.g. self-report, proxy measures) were used in 459 primary studies, included in 20 of the included reviews [13,24,26,27,29–35,37,38,40–46]. Both objective and subjective measurement tools were used by 41 primary studies across 11 reviews [13,24,34,37,38,40–45]. The majority of eligible reviews (n = 25) included ‘Overall PA’ as a primary outcome measures (Table A in S2 Table). In addition to ‘overall PA’, three reviews also included MVPA [28,31,47], and two reviews also included ‘leisure time PA’ as an outcome measure [24,30]. Time-specific PA was included as an outcome within a number of reviews among youth only, such as ‘Recess PA’ [14,35,47] and ‘Afterschool PA’ [35]. PA outcomes, for example, ‘active transport to school’, ‘walking to school’ and ‘cycling to school’ were combined under a single outcome labelled as ‘active transport to school’.

### Categorisation of the included determinants

The physical determinants of PA included in the present umbrella SLR are listed in the Supplementary materials (Table A, B and C in S3 Table). In the preparation phase, a total of 254 physical determinants of PA were identified by extracting reported determinants from the included SLRs and MAs. Amongst these, either duplicates or very close constructs were merged into broader determinants to facilitate the synthesis of findings across all reviews. For example, determinants including ‘*unsuitable weather’*, *‘poor weather’*, *‘adverse weather/climate conditions’* and *‘bad weather’* were combined under a single determinant labelled as *‘weather condition (unfavourable)’*. Given the variation in determinants examined and the definitions employed across reviews, it was not possible to condense determinants in all instances. A final list of 67 physical determinants were included within the SLR (Tables 2, 3 and 4). Determinants were grouped based on the ANGELO framework (i.e. micro or macro environment) and further categorised into specific levels (home, school, neighborhood, city/municipality/region).

**Table 2:** Summary of the results of the included reviews on preschool children: The importance of a determinant and its strength of evidence.

	PA outcome			
Determinant	Overall PA	MVPA	Active Transport	Recess PA
**MICRO-ENVIRONMENT**				
***Home/Household***				
Access/Availability of outdoor toys/objects/equipment	+, Lnc [47]	+, Lnc [47]		
Access/Availability of play/ PA facilities and equipment in the home	00, Lnc [47]			
Access/Availability/Size of backyard space	++, Lnc [47]			
Access/Availability of family transport (own more than 1 car)			0, Pe [47]	
***Educational Institutions***				
Distance to school (<800m)		—, Pe [47]	--, Pe [47]	
Availability of PA equipment/ toys/ play structures in school areas	00, Lnc [48]			00, Lnc [47]
Play space features				00, Lnc [47]
Active means of transport to school	++, Lnc [47]			
***Neighbourhood***				
*Facility availability and accessibility*				
Access/presence of parks/playgrounds/open space	++, Lnc [47,48]	++, Pe [47]		
Distance to PA facilities		--. Lnc [47]		
Access/ Availability of PA infrastructure/ equipment	00, Lnc [47]	00, Lnc [47]		
*Transportation environment*				
Availability/ Access/ Proximity of public transport system			0, Pe [47]	
Negative Street Characteristics			--, Pe [47]	
Presence of street lights		--, Pe [47]		
High traffic density/speed			0, Pe [47]	
Neighbourhood Safety	00, Lnc [48]			
**MACRO-ENVIRONMENT**				
*City/Municipality/Region*				
Season / Temperature	00, Ls [47]			
Weather condition (favourable)	0, Lnc [48]			
Environment aesthetics	00, Lnc [47]			
Rural vs urban school location	++, Lnc [47]	0, Lnc [47]		

(--) all reviews found a negative association; (-) negative association was found in ≥ 75% of reviews/ primary studies; (0) results were mixed, or reported an association in < 75% of available reviews/ primary studies; (00) all reviews reported a null association; (+) positive association was found in ≥75% of the reviews/ primary studies; (++) positive association was found in all reviews. Pe, Probable evidence; Ls, Limited Suggestive; Lnc, Limited, non-conclusive. PA, Physical Activity; MVPA, Moderate-to-vigorous physical activity

**Table 3:** Summary of the results of the included reviews on children and adolescents: The importance of a determinant and its strength of evidence.

	Overall PA (Children)	Overall PA (Adolescents)	Overall PA (Children and Adolescents)	Overall PA (Children and Adults)	MVPA (Children and Adolescents)	Recess/ Afterschool PA	Active Transport to School (Children)	Walking/ cycling during leisure (Children)
**MICRO-ENVIRONMENT**								
***Home/Household***								
Access/ Availability of play/ PA facilities and equipment in the home	0, Lnc [41,49]	0, Pe [41]	0, Ce [38,43]					
Access/Availability/Size of backyard space			0, Lnc [43]					
Access/Availability of family transport	0, Lnc [41,42]		+, Ls [25,45]					
***Educational Institutions***								
***Facility availability and accessibility***								
Distance to school	0, Ls [41,43]		0, Ls [43,44]					
Access/ provision of school facilities/ resources		++, Lnc [41]				0, Lnc [14,35]		
Number of PA programs/activities						++, Lnc [35]		
Access to seating						00, Lnc [35]		
Access to areas that facilitate physical activity*						00, Lnc [35]		
Access to play space						0, Lnc [14,35]		
Access to outdoor obstacle course						0, Lnc [35]		
***Equipment availability***								
Availability of PA equipment/ toys/ play structures in school areas	00, Lnc [41]		+, Lnc [44]			0, Lnc [14,35]		
Access to a gym with cardio & weightlifting equipment						++, Lnc [35]		
***Features of facilities and equipment***								
Play space features						0, Lnc [14,35]		
Condition of facilities						0, Lnc [35]		
Active means of transport to school	0, Lnc [37]	+, Lnc [37]	0, Pe [37,40]		++, Lnc [39]			
Environmental barriers to active travel						00, Lnc [35]		
**Neighbourhood**								
***Neighbourhood design***								
Range of housing opportunities and choice				0, Lnc [34]				
Access/distance/proximity to destinations	—, Lnc [41]		0, Lnc [44]		++, Lnc [39]			
Street characteristics	0, Ls [43]		00, Ls [43]					
Street length	0, Pe [43]		0, Pe [43]					
Negative street characteristics			—, Lnc[44]					
Availability of sidewalks/trails			0 Lnc [44]		++, Lnc [39]			
Street connectivity	0, Lnc [13]	0, Lnc [13]	0, Lnc [44]		++, Lnc [39]		0, Pe [46]	0, Pe [46]
Footpath conditions/ available shelters	00, Lnc [41]		++, Lnc [44]					
Number of roads to cross			0, Lnc [44]					
***Transportation environment***								
Pedestrian and cyclist safety structure	0, Lnc [13]	0, Lnc [13]	++, Lnc [44]					
Presence of Walking and Cycling Paths/Amenities	0, Lnc [13]	0, Lnc [13]	0, Lnc [44]			00, Lnc [35]	0, Pe [46]	0, Pe [46]
Presence of street lights						00, Lnc [35]		
Walkability	++, Lnc [13]	0, Lnc [13]		0, Lnc [34]	++, Lnc [39]		0, Lnc [46]	00, Lnc [46]
Accessibility							0, Ls [46]	++, Ls [46]
Traffic density/speed	0 Ls [13,43]	0, Lnc [13]	0, Pe [43,44]					
Traffic safety							0, Pe [46]	0, Pe [46]
Traffic related hazards	0, Lnc [41]				—, Lnc [39]			
Availability/Access/Proximity of public transport system	0, Lnc [41]		++, Lnc [44]	0, Ls [34]				
***Facility availability and accessibility***								
Access/ proximity parks/playgrounds/open space	0, Lnc [13]	0, Lnc [13]	0, Lnc [44]	0, Lnc [34]	0, Lnc [39]	00, Lnc [35]		
Access/ availability/ proximity recreational facilities	0, Lnc [13]	0, Lnc [13]	+, Lnc [44]				0, Lnc [46]	0, Lnc [46]
Availability/Access/Proximity of PA facilities/programmes	0, Lnc [41]	0, Lnc [41,49]				0, Lnc [35]		
Distance to PA facilities/parks		0, Lnc [41]						
Access/ Availability of PA infrastructure/ equipment	0, Ls [43]	++, Ls [43]	0, Pe [43]			0, Lnc [35]		
Presence of other features (e.g. signage, trees)^						00, Lnc [35]		
***Neighbourhood Safety***								
Neighbourhood Safety	00, Ls [43]		00, Ls [44]			0, Lnc [35]	0, Ls [46]	00, Ls [46]
Crime Safety							0, Ls [46]	0, Ls [46]
Neighbourhood physical disorder	00, Lnc [41]		00, Lnc[44]					
**MACRO-ENVIRONMENT**								
**City/Municipality/Region**								
Population/ residential density	0, Lnc [13]	0, Ls [13]	0, Lnc [44]				0, Lnc [46]	0, Lnc [46]
Weather condition (unfavourable)		0, Ls [41]	0, Lnc [44]					
Season / Temperature	0, Pe [41]	0, Ls [41]	0, Pe [36,44]			0, Lnc [14,35]		
Environment aesthetics		00, Ls [43]	++, Lnc [44]			00, Lnc [14,35]	0, Pe [46]	00, Pe [46]
Vegetation (presence of street trees)	0, Lnc [13]	0, Ls [13]						
Urban vs Rural residential location	0, Lnc [41]	0, Lnc [41]	0, Lnc [44]					
Urban vs Suburban	0, Lnc [41]	0, Lnc [41]			0, Lnc [39]			
Level of urbanization		00, Lnc [41]	0, Lnc [33]					
Land Use Mix Diversity	0, Lnc [13]	0, Lnc [13]		0, Pe [34]		00, Lnc [35]	0, Ls [46]	0, Ls [46]
Urban Form				0, Pe [34]				
Rural school location						++, Lnc [14]		
Coastal location	++, Lnc [41]							

* Access to court space, playing fields, sledding hill, ski tracks, ice-skating areas, fenced courtyard space, climbing wall, a wooded area, water (sea, river, lake), bitumen areas, areas for hopscotch/skipping and areas for boarding/skating, swimming facilities

^ Presence of trees, shade, a water feature, signage regarding dogs, signage restricting other activities

(--) all reviews found a negative association; (-) negative association was found in ≥ 75% of reviews/ primary studies; (0) results were mixed, or reported an association in < 75% of available reviews/ primary studies; (00) all reviews reported a null association; (+) positive association was found in ≥75% of the reviews/ primary studies; (++) positive association was found in all reviews. Ce, Convincing evidence; Pe, Probable evidence; Ls, Limited Suggestive; Lnc, Limited, non-conclusive. PA, Physical Activity; MVPA, Moderate-to-vigorous physical activity

**Table 4:** Summary of the results of the included reviews on adults: The importance of a determinant and its strength of evidence.

	Overall PA (≥ 18 years)	Adults (< 50 years)	Adults (> 50 years)	General walking and cycling	MVPA	Leisure/ Recreational PA	Active Transport
**MICRO-ENVIRONMENT**							
**Neighbourhood**							
***Neighbourhood Design***							
Access/availability/proximity of destinations	0, Lnc [24]			0, Lnc [24]		0, Lnc [24]	0, Lnc [24]
Negative street characteristics	0, Lnc [28,29]						0, Lnc [24]
Street connectivity				++, Lnc [31]		00, Lnc [31]	++, Lnc [31]
***Transportation environment***							
Presence of Walking and Cycling Paths/Amenities	0, Lnc [24,26,28]			0, Lnc [31]	00, Lnc [28,31]	00, Lnc [31]	0, Lnc [24,31]
Presence of street lights	00, Lnc [28]						
Walkability	+, Lnc [24]			0, Lnc [15]		0, Lnc [24]	0, Lnc [15,24]
Availability/ Access/ Proximity of public transport system	00, Lnc [24]	00, Lnc [23]	0, Lnc [23]	++, Lnc [31]	00, Lnc [28]		0, Lnc [24]
Traffic density/speed	0, Lnc [26,28]	0, Lnc [23]	0, Lnc [23]				
Traffic Safety	0, Lnc [24,28]			00, Lnc [24,31]	00, Lnc [28]	0, Lnc [24, 31]	0, Lnc [24,31]
***Facility availability and accessibility***							
Access/ proximity parks/playgrounds/open space	0, Lnc [25]	0, Lnc [23]	0, Lnc [23]	0, Lnc [31]			
Access/ availability/ proximity recreational facilities	0, Lnc [24,28]			0, Lnc [24,31]	00, Lnc [28]	0, Lnc [24, 31]	0, Lnc [24,31]
Non-recreational land use				0, Lnc [31]	00, Lnc [31]	00, Lnc [31]	-, Lnc [31]
Lack of parks and open space		0, Lnc [23]	00, Lnc [23]				
Availability/Access/Proximity of PA facilities/programmes/equipment	00, Lnc [26,28]	0, Lnc [23]	0, Lnc [23]		0, Lnc [28]		
Lack of access to PA equipment/facilities/programmes	0, Lnc [25,29]	0, Lnc [23]	0, Lnc [23]				
Neighbourhood Satisfaction					0, Lnc [28]		
***Neighbourhood Safety***							
Neighbourhood Safety	0, Lnc [24,29]	0, Lnc [23]	0, Lnc [23, 32]	0, Lnc [24]		0, Lnc [24]	
Safety from crime	0, Lnc [24]			00, Lnc [24]		0, Lnc [24]	0, Lnc [24]
**MACRO-ENVIRONMENT**							
**City/Municipality/Region/Country**							
Population/ residential density	0, Lnc [24]			0, Lnc [31]	00, Lnc [31]		
Season/ Temperature						++, Lnc [30]	
Weather condition (unfavourable)	0, Lnc [25,28]	0, Lnc [23]	0, Lnc [23]		00, Lnc [28]		
Air/ Noise Pollution	00, Lnc [48]				00, Lnc [28]		
Environment aesthetics	0, Lnc [24,27,28]			00, Lnc [24,31]	00, Lnc [28]	0, Lnc [24, 31]	0, Lnc [24,31]
Quality of environment	+, Lnc [24]					0, Lnc [24]	
Environment Score	++, Lnc [28]				0, Lnc [28]		
Environmental Barriers	++, Lnc [26]						
Urban vs Rural residential location	++, Lnc [30]						
Level of urbanization	0, Ls [24]	0, Lnc [33]	+, Lnc [33]	0, Ls [24]		0, Ls [24]	+, Lnc [24]
Urban Form	0, Lnc [26,48]			00, Lnc [31]			
Land Use Mix Diversity	0, Lnc [24]			++, Lnc [31]	0, Lnc [28,31]	00, Lnc [31]	++, Lnc [31]
Coastal Location	00, Lnc [28]						

(--) all reviews found a negative association; (-) negative association was found in ≥ 75% of reviews/ primary studies; (0) results were mixed, or reported an association in < 75% of available reviews/ primary studies; (00) all reviews reported a null association; (+) positive association was found in ≥75% of the reviews/ primary studies; (++) positive association was found in all reviews. Ls, Limited Suggestive; Lnc, Limited, non-conclusive. PA, Physical Activity; MVPA, Moderate-to-vigorous physical activity

### Summary of the results of the included reviews by importance of determinants and strength of evidence

The findings of the included reviews are summarized in the supplementary material (Table A in S2 Table), while data on the associations between physical determinants and PA are summarised by stage of the life course; preschool children (Table 2), children and adolescents (Table 3) and adults (Table 4), and further stratified by population (for example, adults < 50years and adults >50 years) and PA outcome where relevant.

#### Preschool children

Of the 31 included reviews, two studied the physical determinants of PA in preschool children (Table 2) [47,48]. To summarise, ‘*access/availability/size of backyard space’* was positively associated with PA at the home level (++, Lnc) [47], while *‘access/availability of outdoor toys/objects/equipment’* was positively associated with overall PA in more than 75% of studies (+, Lnc) [47]. Probable evidence was found for a negative association between ‘*distance to school (<800m)’* and levels of MVPA (—, Pe) [47]; while a positive association was found between ‘*distance to school (<800m)’* and active transport (++, Pe) [47]. *Negative street characteristics’*, including lack of crossings/lights, busy road barriers on the way to school and steep roads on the way to school were all negatively associated with active transport (—, Pe) [47]. ‘*Access/proximity parks/playgrounds/open space’* in the neighborhood was positively related to overall PA in all the studies included in the reviews (++, Lnc) [47,48]. A positive association was also observed for MVPA, with a probable level of evidence (++, Pe) [47]. Attending a preschool in a rural area (‘*rural vs urban school location’)* was positively associated with overall PA (++, Lnc) [47].

#### Children

Among the 31 included reviews, seven explored the physical determinants of PA in children [13,37,41–43,46,49] (Table 3). At the home level, inconclusive results were found for ‘*access/availability of play/PA facilities and equipment in the home’* (0, Lnc) [41,49] and ‘a*ccess/availability/proximity to family transport’* (0, Lnc) [41,42]. *‘Availability of PA equipment/toys/play structures in school areas’* was not associated with PA in children (00, Lnc) [41]. At the neighbourhood level, *‘access/distance/proximity to destinations’* was negatively associated with overall PA (—, Lnc), while *walkability* was positively associated (++, Lnc) [13]. General ‘a*ccessibility’* at the neighborhood level was positively associated with walking/cycling during leisure time, with a limited, suggestive level of evidence (++, Ls) [46].

#### Adolescents

Five reviews studied the physical determinants of overall PA among adolescents [13,37,41,43,49] (Table 3). Inconsistent associations were reported for *‘access/availability of play/PA facilities and equipment in the home’* and overall PA (0, Pe) [41]. ‘*Access/provision of school facilities/resources’* was positively associated with overall PA in adolescents [41], (++, Lnc). In contrast to the inconsistent findings observed for ‘*active means of transport to school’* among children, a positive association was reported for more than 75% of the studies included in the review for adolescents (+, Lnc) [37]. ‘*Access/availability of PA infrastructure/equipment’* in the neighborhood was positively associated with overall PA in adolescents [43], with a limited, suggestive level of evidence (++, Ls). The majority of reported associations in adolescents were inconsistent.

#### Children and adolescents

Eleven of the included reviews reported associations on physical determinants in children and adolescents combined [14,35–40,43–45,50] (Table 3). ‘*Access/availability of family transport’*, defined as parents providing support through transportation, was positively associated with overall PA in more than 75% of the studies included in the review (+, Ls) [45,50]. ‘*Availability of PA equipment/toys/play structures in school areas’* was positively associated with overall PA in more than 75% of the studies included in the review (+, Lnc) [44]. An inconsistent associations between *‘active means of transport to school’* and overall PA was found within included reviews (0, Pe) [37,39,40]. ‘*Number of PA programs/activities’* and *‘access to a gym with cardio & weightlifting equipment’* within the school setting were positively associated with recess/afterschool PA (++, Lnc) [35]. At the neighborhood level, presence of ‘*negative street characteristics’* for example, steep terrain, [44] and *‘traffic related hazards’* for example, nearby roads and intersections, [39] were negatively associated with overall PA (—, Lnc) and MVPA respectively (—, Lnc). Inconclusive evidence was found for ‘s*eason/temperature’* and overall PA [36,44], although the level of evidence was probable (0, Pe). Higher ‘e*nvironment aesthetics’* i.e. having more interesting things to look at [44] was positively associated with overall PA (++, Lnc).

#### Adults and older adults

The physical determinants of PA among adults were investigated by eleven reviews [15,23–32] (Table 4). At the neighborhood level, a negative association was reported for ‘*negative street characteristics’*. For example, lack of sidewalks and street lights [29] and overall PA in more than 75% of the studies included (-, Lnc). ‘*Walkability’* [24] was positively associated with overall PA in more than 75% of the studies included in the review with a limited, non-conclusive level of evidence (++, Lnc). At the macro level, ‘*environment score’* [28], ‘*environmental barriers’* [26] and ‘*urban vs rural residential location’* [30] were all positively associated with overall PA (++, Lnc). ‘*Street connectivity’* and ‘*land use mix diversity’* were positively associated with active transport in adults [31] (++, Lnc), while *‘street connectivity’*, *‘availability/access/proximity of public transport system’* and ‘*land use mix diversity’* were positively associated with general walking and cycling [31] (++, Lnc). *Season/temperature* was positively associated with leisure/recreational PA in adults [30] (++, Lnc). The majority of reported associations between physical determinants and general walking and cycling, leisure/recreational PA and active transport were inconsistent (Table 4).

#### Whole population

One review examined the association between *‘level of urbanisation’* and overall PA across the life course [33]. Inconsistent associations were reported in children and adolescents (0, Lnc) [33], while *‘level of urbanisation’* was positively associated with overall PA in more than 75% of the studies in adults (+, Lnc). One review reported associations on physical determinants of overall PA irrespective of the age of the participants [34]. Inconclusive associations were reported for ‘*range of housing opportunities and choice’*, *‘walkability’* and ‘*access/ proximity parks/playgrounds/open space’* (0, Lnc) [34], and *‘availability/access/proximity of public transport system’* (0, Ls) [34]. A probable level of evidence was reported for ‘*urban form’* and ‘*land use mix diversity’;* although reported associations were inconsistent (0, Pe) [34].

### Quality assessment

The quality assessment based on the AMSTAR checklist was performed for the 28 included SLRs (Table 5). The majority of reviews (n = 21) were found to be of moderate quality (4–7 points), with 9 reviews of weak quality (2–3 points) [24,26,30,34,36–38,49,50]. The majority of reviews conducted a comprehensive literature search (n = 27) and reported characteristics of the included primary studies within the review (n = 25). The scientific quality of the included primary studies was assessed and documented in 10 reviews [23,25,29,32,33,35,38,40,43,46]. One reviews assessed the likelihood of publication bias [32], while none of the included SLRs or MAs provided a list of both included and excluded primary studies.

**Table 5:** Quality assessment of the included systematic literature reviews using the AMSTAR checklist.

Author, Date [Ref]	Was an 'a priori' design provided?	Duplicate study selection and data extraction	Comprehensive literature search	Status of publication used as an inclusion criterion	List of studies (included & excluded) provided	Characteristics of included studies provided	Scientific quality of included studies assessed and documented	Scientific quality used appropriately in formulating conclusions	Appropriate methods to combine the findings of studies	Likelihood of publication bias assessed	Conflict of interest included	Sum quality score*	Quality of the review**
**Babacus, 2012 (SLR)**[25]	No	Yes	Yes	Yes	No	Yes	Yes	No	Yes	No	No	6	MOD
**Beet, 2010 (SLR)** [50]	Yes	C.A	Yes	No	No	No	No	C.A	C.A	No	Yes	3	WEAK
**Casagrande SS, 2009 (SLR)** [26]	Yes	Yes	No	No	No	Yes	No	N.A.	N.A.	No	No	3	WEAK
**Coble JD, 2006 (SLR)**[27]	No	No	Yes	Yes	No	Yes	No	No	Yes	No	No	4	MOD
**Craggs C, 2011 (SLR)**[43]	Yes	Yes	No	No	No	Yes	Yes	Yes	N.A.	No	Yes	6	MOD
**Davison KK, 2006 (SLR)** [43]	Yes	Yes	Yes	Yes	No	Yes	No	No	Yes	No	No	6	MOD
**De Craemer M, 2012 (SLR)**[47]	Yes	Yes	Yes	No	No	No	No	N.A.	N.A.	No	Yes	4	MOD
**Ding D, 2011 (SLR)** [13]	Yes	Yes	Yes	Yes	No	Yes	No	No	N.A.	No	Yes	6	MOD
**Durand CP, 2011 (SLR)** [34]	No	Yes	Yes	No	No	Yes	No	N.A.	N.A.	No	No	3	WEAK
**D'Haese S, 2015 (SLR)** [46]	Yes	C.A.	Yes	No	No	Yes	Yes	Yes	Yes	No	Yes	7	MOD
**Ferreira I, 2007 (SLR)** [41]	Yes	No	Yes	Yes	No	Yes	No	No	No	No	Yes	5	MOD
**Gustafson SL, 2006 (SLR)** [42]	Yes	C.A	Yes	N.A.	No	Yes	No	Yes	Yes	N.A.	Yes	6	MOD
**Hinkley T, 2008 (SLR)**[48]	Yes	Yes	Yes	N.A.	No	No	No	No	N.A.	No	Yes	4	MOD
**Koeneman MA, 2011 (SLR)** [32]	No	Yes	Yes	No	No	Yes	Yes	Yes	C.A.	Yes	Yes	7	MOD
**Lachowycz K, 2011 (SLR)** [33]	No	No	Yes	No	No	Yes	Yes	Yes	Yes	No	Yes	6	MOD
**Larouche R, 2014 (SLR)** [40]	Yes	Yes	Yes	Yes	No	Yes	Yes	Yes	N.A.	No	No	7	MOD
**Lee MC, 2008 (SLR)** [37]	Yes	No	Yes	No	No	Yes	No	No	No	No	No	3	WEAK
**Maitland C, 2013 (SLR)** [38]	No	No	Yes	No	No	Yes	Yes	No	N.A.	No	No	3	WEAK
**McCormack GR, 2011 (SLR)** [31]	No	Yes	Yes	No	No	Yes	No	No	N.A.	No	Yes	4	MOD
**Olsen JM, 2013 (SLR)** [29]	Yes	No	Yes	No	No	Yes	Yes	No	N.A.	No	C.A	4	MOD
**Rich C, 2012 (SLR)** [36]	Yes	No	Yes	No	No	C.A	No	No	N.A.	No	No	2	WEAK
**Ridgers ND, 2012 (SLR)** [14]	Yes	C.A	Yes	No	No	Yes	No	N.A.	N.A.	N.A.	Yes	4	MOD
**Siddiqi Z, 2011 (SLR)** [23]	Yes	No	Yes	No	No	Yes	Yes	Yes	N.A.	No	Yes	6	MOD
**Stanley AM, 2012 (SLR)** [35]	No	Yes	No	No	No	No	Yes	Yes	N.A.	No	Yes	4	MOD
**Tzormpatzakis N, 2007 (SLR)** [30]	No	C.A	Yes	No	No	Yes	No	C.A	N.A.	No	No	2	WEAK
**Van der Horst K, 2007 (SLR)** [49]	No	Yes	Yes	No	No	Yes	No	N.A.	N.A.	No	No	3	WEAK
**Van Holle V, 2012 (SLR)** [24]	No	No	Yes	No	No	Yes	No	N.A.	N.A.	No	No	2	WEAK
**Wendel-Vos W, 2007 (SLR)** [28]	No	Yes	Yes	No	No	Yes	No	No	No	No	Yes	4	MOD

C.A., Can't answer; N.A., Not applicable; MOD, Moderate,

*0 when the criteria was not applicable for the included review; 1 when the criteria was applicable for the included review.

**Weak (score ranging from 0–3); Moderate (score ranging from 4–7); Strong (score ranging from 8–11). AMSTAR checklist used to appraise systematic literature reviews only, not included meta analyses.

## Discussion

### Summary of evidence

This umbrella SLR summarised the current research on the physical determinants of PA across the life course, identifying 67 determinants from 31 reviews relating to the physical components of the broader environment level of determinants of PA. The majority of reviews within this umbrella SLR focused on determinants of PA in youth (from preschool to adolescents) (n = 18). Amongst preschool children *‘access/availability/size of backward space’* was positively associated with overall PA, while *‘negative street characteristics’* were negatively associated with active transport. Similarly, *‘negative street characteristics’* were negatively associated with overall PA in children and adolescents. At the school level, ‘*number of PA programs/activities’* and *‘access to a gym with cardio & weightlifting equipment’* were positively associated with recess/afterschool PA in children and adolescents. In adults, consistent positive associations were found for *‘walkability’* and overall PA, while *‘street connectivity’* and *‘land use mix diversity’* were positively associated with active transport.

Physical determinants of PA at the home level were only explored in studies involving youth (<18 years). In preschool children, *‘access/availability/size of backyard space’* and *‘access/availability of outdoor toys/objects/equipment’* were positively associated with PA [47]. The home environment is a key influence on PA at this stage of the life course [51], particularly for preschool children who have limited independent mobility and spend the majority of their time within the home setting [38]. Inconclusive results were observed for *‘access/availability of play/ PA facilities and equipment’* in the home in children and adolescents [41,49], suggesting that equipment in the home is more important for preschool children’s PA, which is typically unplanned and unstructured [48]. Inconsistent associations were observed for *‘Access/availability of family transport’* in preschool children; however, this determinant was positively associated with overall PA in more than 75% of the studies in children and adolescents [45,50]. The positive association observed in older children and adolescents suggests that instrumental support, for example, access to family transport to participate in other types of PA outside the home environment, for example, at the school or neighborhood level, is more important than access to PA equipment within the home in this age group.

Within the educational setting, *‘availability of PA equipment/ toys/ play structures in school areas’* was not associated with overall PA in preschool children; however, a positive association was observed in more than 75% of studies in children and adolescents [44]. Time-specific PA across the school day was also explored within the present SLR, with the *‘number of programs/activities available’* and *‘access to a gym with cardio & weightlifting equipment’* both positively associated with recess/afterschool PA in children and adolescents. Recess has been shown to make a valuable contribution to children’s PA across the school day [52]; however, the evidence highlighted within the present SLR was drawn from a limited number of cross-sectional studies. *‘Access/provision of school facilities/resources’* was positively associated with overall PA in adolescents, highlighting the importance of the school setting in providing an environment where adolescents can engage in PA. Given the age related decline observed in PA levels across adolescence [53], the school setting may provide a suitable environment for promoting PA, providing the appropriate facilities and resources are available.

In addition to the associations observed within the school day, the present SLR also identified a number of determinants associated with active transport to school. Amongst children attending preschool, a positive association was found between *‘distance to school (<800m)’* and active transport [47]. *‘Negative street characteristics’*, including no lights/crossings and busy road barriers on the way to school were negatively associated with active transport [47], highlighting that features within the physical environment may need to be modified to encourage this type of PA behavior. An *‘active means of transport’* to educational institutions was not associated with overall PA in preschool children [47], with inconsistent associations reported within the included reviews amongst children [37,39,40]. In contrast, a positive association was reported for more than 75% of the studies included in the review for adolescents [37]. Active transport has been readily cited as a contributor to habitual PA in youth [54], and may be particularly important to address the declining levels of PA amongst adolescent females [55], with evidence highlighting a greater association between active transport and MVPA in females of this age range [55]. Given that the school environment, and a ‘whole of school approach’, has been identified as one of the key investments for the promotion of PA [56], the determinants identified as potentially influencing overall PA and PA across the school day within the present umbrella SLR should be further examined in this population.

The neighborhood environment has the potential to influence PA across childhood, providing opportunities for both structured, planned PA and incidental bouts of PA [57]. Within the present umbrella SLR, *‘access/proximity parks/playgrounds and open space’* was positively associated with preschool children’s overall PA and MVPA [47,48]. These findings suggest that the provision and proximity of areas for recreational play, such as playgrounds may have an impact on PA at this stage of the life course, and should be considered within polices for neighborhood design. Although inconsistent associations were observed for *‘access/proximity parks/playgrounds/open space’* and overall PA in children and adolescents [13,44], *‘access/availability of PA infrastructure/ equipment’* within the neighborhood was positively associated with overall PA in adolescents [43]. These observed associations further reinforce the need for schools and communities to provide adequate resources to facilitate PA behaviors in adolescents.

Recreational opportunities for PA represent a dominant domain of PA in children [58] therefore policy makers and urban planners should ensure opportunities for PA within the neighborhood are both child and activity friendly. Walkability was positively associated with overall PA in children [13] and MVPA in children and adolescents [39]. In conjunction with this, a number of determinants were shown to be negatively associated with PA, including *‘negative street characteristics’* and *traffic related hazards’*. These findings highlight that features at the neighborhood level can both promote and inhibit PA behaviors in children and adolescents. The identification of a number of modifiable determinants within this SLR, such as improving traffic safety, highlights a potential for neighborhoods to make small improvements, which may contribute to increased levels of PA [59].

Given that PA behaviours in youth are influenced by factors at a number of different levels; including the home, school and neighbourhood environment [41], it is important that future interventions target all of these levels and also consider elements of the macro environment in order to effectively address the problem of physical inactivity in youth [41]. The variation across reviews on the determinants evaluated hampers the conclusions that can be drawn. Determinants studied in preschool children were supported by, at most, two reviews, and in children and adolescents three reviews.

Within the present SLR, physical determinants of PA among adults were identified at the neighborhood level and the macro level. *‘Walkability’* [24] was positively associated with overall PA; however, this association was not consistently reported across all studies. Inconsistent associations were reported between walkability and other outcomes, including general walking and cycling, leisure/recreational PA and active transport. The impact of neighborhood walkability on PA has been quantified in a number of studies, with those living in highly walkable neighborhoods undertaking 50 minutes more of walking for transport a week compared to those in less walkable neighborhoods [60]. The inconsistent associations observed within the present umbrella review may be attributable to the variability in measurement tools and concepts used to define walkability [15]. Furthermore, other features at the neighborhood level, for example, safety and social cohesion may influence the relationship between walkability and PA, and should therefore be considered in future studies [15]. ‘*Street connectivity’* and *‘land use mix diversity’* were positively associated with active transport and general walking/cycling in adults [31], *‘availability/access/proximity of public transport system’* was also positively associated with general walking/cycling [31]. Distinguishing what features within the physical environment influence specific types of PA in adults is important to advance knowledge in the field, as these identified determinants can be modified within the quasi-experimental setting to further evaluate their contribution to PA. Evidence has shown that adults who engage in active travel have significantly higher total PA compared with those who do not [61] Therefore, enhancing opportunities for active travel within neighborhoods may present a valuable opportunity to promote PA in this population.

*‘Season/temperature’* was positively associated with leisure time PA in adults yet it was only assessed in one review [30], while inconsistent associations were reported for *‘weather condition (unfavourable)’* and overall PA [23,25,28]. Given that both season and weather should be controlled for within studies examining PA [62], it is surprising that so few studies examined the associations between season/weather conditions and PA within the present SLR. Establishing how such environmental factors influence PA, particularly across different regions and countries, and in high risk populations, is important in the development of future interventions to overcome perceived barriers to PA, of which poor weather is often cited [62]. A number of other determinants at the macro-environment level were positively associated with overall PA, with those living in rural areas more likely to be physically active than their urban counterparts [30].

A number of determinants within the physical environment were found not to be associated with overall PA in adults, including *‘presence of streetlights’* [28], *‘availability/access/proximity of public transport systems’* [24], ‘*availability/ access/ proximity of PA facilities/programmes/equipment’* [26,28] ‘*air/noise pollution’* and *‘coastal location’* [28]. The majority of reported associations between physical determinants and general walking and cycling, leisure/recreational PA and active transport were inconsistent, limiting conclusions on how physical determinants are associated with these domains of PA in adults. The majority of evidence was drawn from cross-sectional studies and was limited, non-conclusive. Therefore, more rigorous study design is needed to question the lack of associations observed for these physical determinants on PA in adults.

This umbrella SLR did not identify SLRs or MAs that included primary studies exploring the relationship between physical environment determinants at the household or workplace level and PA behaviours in adults. The workplace has been a focal point for numerous behaviour change interventions aimed at promoting PA in adults [63]. To date, findings on the effectiveness of interventions in the workplace setting are inconclusive [63]. Longitudinal and cross-sectional studies are needed to determine what factors within the physical environment are associated with PA, which can then inform the design of future workplace interventions. Finally, the discrepancies in the associations between the environmental features and PA might be attributable to the perceived vs. objectively measured nature of the environmental feature, the geographical definition of the exposure area (i.e. census tract vs. home centred buffers), the definition of the variable itself (unique indicator vs. index), and varying quality of data between studies. Additionally, the diversity of countries/cities of studies increases the difficulties in being able to compare estimates, even after controlling for confounding factors.

### Limitations

In addition to the limitations reported within the individual SLRs and MAs (Table 5), the present SLR has highlighted a number of limitations within the literature which need to be considered in the design and implementation of future studies in this field. The variation across included SLRs and MAs, and the primary studies included within them in terms of population studied, measurement techniques and the PA outcomes assessed resulted in limited comparisons and conclusions within this umbrella SLR. The majority of included primary studies relied on self-report methods to assess PA outcomes and determinants, which may impact upon the associations observed. In addition to variation across studies on PA measurement, determinants in the physical environment were defined and measured differently across studies. Lack of detail within included reviews limited how certain determinants could be defined within the present umbrella SLR. The majority of evidence was drawn from cross-sectional studies and was limited, not conclusive therefore more rigorous study design is needed to confirm any observed associations with this umbrella SLR.

Given the scope of the physical environment, encompassing ‘what is available’ [10], it is not surprising that the present umbrella SLR initially identified 254 variables relevant to the physical environment. To facilitate the synthesis of results and provide a succinct overview, it was necessary to combine variables and related PA outcomes into sub-groups. While efforts were made to ensure only similar variables and PA outcomes were grouped together, it is possible that some detail may have been lost. In some instances, the lack of detail or explanation provided for variables within primary studies, particularly in relation to the direction of the association, limited the explanation certain associations observed within the present umbrella SLR. For example, associations were observed for *‘residential density’* but it was not always possible to distinguish if this association related to high or low residential density. Furthermore, given the wide range of determinants investigated across different stages of the life course, the same set of determinants were not consistently reviewed across all included studies, which limits the analysis of results [43]. The majority of included SLRs and MAs did not assess the scientific quality of their included primary studies. In addition, there is currently a lack of consensus on how to grade the evidence of individual SLRs and MAs within an umbrella review. To overcome this limitation, the methods employed within this SLR were based on those used in a previous umbrella review, which will increase the comparability of findings with other umbrella reviews in the field [21].

### Conclusions

This is the first review to integrate findings from previous reviews and provide an overview of physical determinants of PA across the life course. Among youth, a number of associations were identified for determinants across all levels of the micro-environment, emphasising the importance of policies and interventions that encompass all aspects of the physical environment young people are exposed too. Although fewer reviews focused on adult populations, a number of key determinants, including street characteristics, walkability and land use mix, were associated with PA. Given that the majority of associations were based on cross-sectional studies, future studies should examine the associations highlighted within this review in studies that are longitudinal in design, which will subsequently inform behaviour change interventions. It is important that consistent definitions for both PA and determinants within the physical environment are employed within future studies examining associations, to facilitate the pooling and harmonisation of future reported associations. In addition, the use of objective measures of PA and the physical environment should be made a priority in order to improve the quality of evidence.

## Supporting information

S1 Table Search strategy and key words used for the literature research. (PDF)

S2 Table Outcomes, determinants and results of included reviews. (PDF)

S3 Table Categorization of extracted potential physical environmental determinants. (PDF)

S4 Table PRISMA 2008 checklist. (DOC)
