# Supplementary material for: A life course examination of the physical environmental determinants of physical activity behaviour: A “Determinants of Diet and Physical Activity” (DEDIPAC) umbrella systematic literature review
Source: PLoS One. 2017 Aug 7;12(8):e0182083. doi: 10.1371/journal.pone.0182083 (PMC5546676; doi:10.1371/journal.pone.0182083)
Supplement: S1 Table — (PDF) [file pone.0182083.s001.pdf]

Supplemental Material

S1 Table: Search strategy and key words used for the literature research.

Set	Search terms
#1	“physical activit*” OR “physical exercise*” OR sport OR “motor activit*” OR “locomotor activit*” OR athletic* OR fitness OR “physical movement*” OR “ physical performance*” OR “aerobic exercise*” OR “physical effort*” OR “physical exertion*”
#2	determinant OR determinants OR correlator OR correlators OR mediator OR mediators OR moderator OR moderators OR contributor OR contributors OR factor OR factors OR association OR modifier OR modifiers OR confounder OR confounders OR pattern OR patterns OR predictor*
#3	demographic* OR motivation OR cognition OR emotion* OR attitude* OR “self-perception” OR “self-confidence” OR “self-efficacy” OR competence OR reward* OR success* OR challenge* OR knowledge OR belief* OR “personal trait*” OR “body image” OR satisfaction OR “time availability” OR “perceived environment” OR family OR peer* OR school* OR leader* OR coach* OR group* OR “climate” OR network* OR employment OR retirement OR “educational level” OR SES OR “socioeconomic status” OR “local identity” OR “national identity” OR value* OR tradition* OR “social expectation*” OR “social trend*” OR “social barriere*” OR “availability of tool*” OR “availability of service*” OR “access to tool*” OR “access to service*” OR neighborhood OR “community route*” OR “school environment” OR “work environment” OR architecture OR urbanization OR transport OR traffic OR “facilit* in public space*” OR advertisement OR “availability of sport club*” OR “availability of fitness center*” OR advocacy OR lobbying OR “corporate social responsibility” OR “physical activity promotion initiative*” OR legislation OR health OR education OR tourism OR environment OR “urban planning” OR transport* OR sport OR sports OR culture OR dance OR theater OR “gender mainstreaming” OR “social inclusion” OR “fiscal measure*” OR program* OR plan OR plans OR communication OR media OR guideline*
#4	“systematic literature review” OR “meta-analysis”
