# Supplementary material for: A life course examination of the physical environmental determinants of physical activity behaviour: A “Determinants of Diet and Physical Activity” (DEDIPAC) umbrella systematic literature review
Source: PLoS One. 2017 Aug 7;12(8):e0182083. doi: 10.1371/journal.pone.0182083 (PMC5546676; doi:10.1371/journal.pone.0182083)
Supplement: S2 Table — (PDF) [file pone.0182083.s002.pdf]

S2 Table: Outcomes, determinants and results of included reviews

Author, Date (Type of review) [Ref]	Outcome (s)	Determinant(s)	Review aim	Overall qualitative results of the review	Overall quanti-tative results of the review	Overall limitations of the study	Overall Recommendations
**Babacus WS, 2012 (SLR)[25]**	Overall PA	Weather condition; Access/ proximity parks/playgrounds /open space; Lack of access to PA equipment/ facilities/ programmes	To assess what is known about levels PA and sedentary time; to contextualize these behaviours among South Asian women with an immigrant background	Lack of access to culturally appropriate facilities and open space, and weather were all barriers to PA in South Asian women.	N.A.	Quantitative results for physical determinants were not included; lack of detail from some of the included papers; possibility of publication and researcher bias; significant heterogeneity among studies.	More research should be dedicated to standardize objective PA measurement and to understand how to utilize the resources of the individuals and communities to increase PA levels and overall health of South Asian women
**Beets MW, 2010 (SLR) [50]**	Overall PA	Access/ Availability to family transport	To explore the provision of social support by parents and its relation to youth PA levels	Parental provision of transportation to places where young people can be active was associated with higher levels of PA in youth.	N.A.	Inconsistencies within the literature on what social support contains makes it difficult to compare findings. Use of different PA methods between studies impacts upon findings.	An inventory of the social support for PA currently provided by parents should be assessed to enable the identification of areas where parents should place increased emphasis for PA support. Given that mothers and fathers may influence PA differently, it is important that such factors are studied.
**Casagrande SS, 2009 (SLR) [26]**	Overall PA	Availability of sidewalks/trails; Traffic density/ speed; Availability/ Access/ Proximity of PA facilities/ programmes; Environmental	To investigate the built environment and its association with PA, diet, and obesity among African Americans	Light traffic and the presence of sidewalks were more often positively associated with PA, although associations were not consistent.	N.A.	Given the small number of included studies, it was not possible to conduct a meta-analysis. Lack of consistency between studies on terminology employed and methods used.	There is a need for consistent terminology and measures when studying the built environment. More studies need to include objective measures of the built environment. Longitudinal studies are needed to identify causal associations.

		barriers of PA; Urban form					
**Coble JD, 2006 (SLR)[27]**	Overall PA	Environment aesthetics	To explore the PA behaviours of Native Americans from the United States and Canada	Inconsistent or indeterminate results were found for physical environmental determinants. These results may be partly due to the paucity of research.	N.A.	The assessment tools used within some individual studies were not validated in Native-American populations. The review was limited to published research.	Given that Native Americans tend to rely on oral traditions to communicate, the use of qualitative studies may help inform the development of quantitative tools to use in this population. Longitudinal studies are needed in the area.
**Craggs C, 2011 (SLR)[43]**	Overall PA	Distance to school; Access/ Availability of PA infrastructure/ equipment; Street length; Street characteristics; Traffic density/speed; Neighbourhood safety; Environment aesthetics	To systematically review the published evidence regarding determinants of change in PA in children and adolescents	Evidence suggests there may be a tendency toward an association between smaller declines in PA and road length and traffic-regulating features however conclusions cannot be drawn based on the limited evidence available.	N.A.	Variation between studies in relation to sample and outcome measures assessed limited interpretation; findings focus on the direction of association, as opposed to the strength of the association; number of studies drawn from the same cohort which may introduce bias.	Future studies need to investigate all domains of the ecological model and use objective measures of PA/ validated questionnaires. Studies should target understudied populations (younger children, adolescents). Further research needed to build the evidence base in relation to environmental determinants, which should be assessed both objectively and subjectively.

**Davison KK, 2006 (SLR) [44]**	Overall PA	Home Equipment; Access/ proximity parks/playgrounds /open space; Access/ availability/ proximity recreational facilities; Distance to school; Equipment/play structures in school play areas; Availability of sidewalks/trails; Footpath conditions/ available shelters; Presence of Walking and Cycling Paths; Presence of controlled crossings; Street Connectivity; Access/availability/proximity of destinations; Availability/ Access/ Proximity of public transport system; Number of roads to cross; Traffic density/speed; Pedestrian and cyclist safety structure; Street	To examine the association between children's PA and environmental attributes (perceived and objectively measured)	Children’s PA participation was positively associated with publicly provided recreational infrastructure and transport infrastructure (presence of sidewalks and controlled intersections, access to destinations and public transportation). Local conditions and transport infrastructure (number of roads to cross and traffic density/speed) were negatively associated with children's PA participation.	N.A.	Restricting the search to studies published in English or searchable in English-based databases may have excluded potentially relevant studies; possibility of publication bias; limited research focused on youth populations outside of the health sciences.	Future research needs to assess both objective and perceived measures of the physical environment, alongside objective measures of PA. Studies should adopt a longitudinal design and examine the key role parents play in children’s use of the physical environment. Research outside of the US should be a focus for future studies to determine whether results identified using US samples can be generalised internationally.

		characteristics; Neighbourhood safety; Physical disorder/tidiness of area; Environment aesthetics; Season/ Temperature; Weather condition; Urban vs Rural residential location; Population/ residential density					

**De Craemer M, 2012 (SLR)[47]**	Overall PA; MVPA; active transportation; PA during recess.	Environment aesthetics; Access/ proximity parks/playgrounds /open space; Preschool attended (rural/urban); Access/ Availability of play/ PA facilities and equipment in the home; Access/Availability to family transport; Active means of transport to school; Outdoor equipment; Access/Availability/Size of backyard space; Access/ Availability of PA infrastructure/ equipment; Distance to school (<800m); Availability of PA equipment/ toys/ play structures in school areas; Play space condition; Markings; Green spaces Distance to PA facilities/parks; Number of	To systematically review the correlates of PA, sedentary behaviour and eating behaviour in children aged 4-6 years	Convenient play spaces, higher frequency of spending time in play spaces, attending a rural preschool, active transport to school, outdoor variables (open space, fixed equipment and wheel toys), weekdays and a big backyard were correlated with higher levels of PA.	N.A.	Some limitations regarding the coding of the association of the variables; some variables were included in summary tables despite only one comparison being available (other studies include at least 3).	Future studies should investigate similar correlates of all PA behaviours to develop more efficient interventions. Strategies should target both genders, all ethnic groups and a range of social classes. A particular focus should be given to promoting PA at the weekend.

		recreational facilities in public open spaces; Availability/ Access/ Proximity of public transport system; Presence of street lights; Lack of street lights; Street Characteristics; Busy road barrier; Traffic density/speed; Season / Temperature					
**Ding D, 2011 (SLR) [13]**	Overall PA	Access/ proximity parks/playgrounds /open space; Access/ availability/ proximity recreational facilities; Street connectivity; Walkability; Pedestrian/cycling amenities; Pedestrian and cyclist safety structure; Traffic density/speed; Vegetation; Land use mix diversity;	To examine the association between environmental attributes and physical activity among youth	In children, walkability, traffic speed/volume, access/proximity to recreation facilities, land-use mix, and residential density were consistently associated with PA. The most supported correlates for adolescents were land-use mix and residential density. The measurement tools used to assess both environmental attributes and PA	N.A.	Results did not take into account effect size therefore conclusions cannot be drawn on magnitude of the associations reported. Associations may vary between sub-groups however this analysis was not conducted. The vote count review method gave same weight to studies with varying sample size therefore results may be influenced by a large number of studies with small sample size.	Future studies should include both objective and perceived measures, using consistent definitions and validated measurement tools. It is important that studies provide methodologic details regarding the operationalization of environmental constructs to facilitate the synthesis of findings.

		Population/residential density		greatly influenced associations.			
**Durand CP, 2011 (SLR) [34]**	Overall PA	Access/ proximity parks/playgrounds /open space; Range of Housing Opportunities and Choices; Walkability; Land use mix diversity; Availability/ Access/ Proximity of public transport system; Urban Form	To investigate whether built environment factors that have been used in smart growth planning are associated with PA or body mass	Significant associations were found between PA and one principle of smart growth (open space preservation). In relation to walking specifically, a range of housing choices, mixed land use and compact building design. Results varied by gender and methods used to assess environmental factors.	N.A.	Difficulties in mapping data from included studies with the smart growth principles meaning subjective judgements had to be made; difficult to distinguish whether principles studied were pre-existing features of the built environment or whether they had been implemented as a result of smart growth planning.	Further research needs to include a wider distribution of races and genders to increase the generalisability of findings. Studies should include both objective and subjective measures of the built environment. Future smart growth community planning should include a focus on health outcomes to allow researchers to explore what principles of smart growth produce the greatest benefits in relation to health.
**D'Haese S, 2015 (SLR) [46]**	Active Transport to School, Walking to School, Cycling to School, Walking during leisure, Cycling during leisure,	Walkability; Street connectivity; Accessibility; Pedestrian/cycling amenities; Access/ availability/ proximity recreational facilities; Neighbourhood safety; Crime	To determine the relationship between a wide range of physical environmental characteristics and different contexts of active transportation in 6- 12 year old children across different continents	Active transportation to school (walking and/or cycling) was positively associated with walkability. No convincing evidence was found for associations between active transportation during leisure and	N.A.	Possibility of publication bias; other non-physical determinants may also influence active transportation in youth; giving the same weight to all results may have influenced findings, for example, studies with small sample sizes or studies that reported many results may have	Given that most of the findings were drawn from cross-sectional data, there is a need for more longitudinal studies. Furthermore, there is a need for studies conducted outside of the USA and Europe, for example, in Asia, Africa and South-America.

	Walking/cycling during leisure	safety; Traffic safety; Environment aesthetics/quality; Land use mix diversity; Population/ residential density		the physical environment. Geographical differences were observed, for example, general safety and traffic safety were associated with active transportation to school in North America and Australia but no association was found in European populations.		strongly influenced overall findings.	
**Ferreira I, 2007 (SLR) [41]**	Overall PA	Access/ availability family transport; Access/ Availability of play/ PA facilities and equipment in the home; Distance to school; Availability of PA equipment/ toys/ play structures in school areas; Access/ provision of school facilities/ resources; Access/availability/proximity of destinations; Availability/Access/Proximity of	To review and update the evidence on correlates of PA in youth, focusing specifically on environmental correlates.	Neighbourhood safety was associated with higher PA in adolescents. Convincing evidence for the role for many other environmental factors was lacking. Some associations were not possible to infer from the limited number of existing studies (particularly those at the neighbourhood/school level as well as at the macro level).	N.A.	Search methodology may have excluded potentially relevant studies; Variations in the measurement of PA across settings made it difficult to determine the specific environmental correlates of specific types of PA.	Future research should investigate the environmental correlates of PA using a longitudinal/ experimental design. To advance the field, it is important that studies adopt clear, standardised definitions and measurement of both environmental variables and PA.

		PA facilities/programmes; Distance to PA facilities; Footpath conditions/ available shelters; Traffic safety; Neighbourhood physical disorder; Availability/ Access/ Proximity of public transport system; Urban vs Rural residential location; Urban vs. suburban; Level of urbanization; Coastal location; Season/ Temperature; Weather condition					
**Gustafson SL, 2006 (SLR) [42]**	Overall PA	Access/Availability to family transport	To unite the existing research on parental influences on children's PA behaviours in order to establish direction for future research and improve existing intervention programmes.	Significant correlations were found between parental support and child PA levels. Facilitation of PA (including transporting the child to exercise/ sporting venues or providing equipment, access, or opportunities to be active) has been strongly correlated with children's PA	N.A.	Conclusions drawn from cross-sectional and longitudinal studies; Lack of standardised methods across studies.	Future research should use experimental design to increase the certainty of results on well established relationships, for example, parental support and child PA.

				levels.			
**Hajna S, 2015 (MA) [15]**	General walking, transportation walking	Walkability	To summarize the current body of knowledge on the association between neighbourhood walkability and total walking in adults	Higher walkability was conclusively associated with a greater number of daily steps in three of the six included studies. Three of the six studies assessed the role of walkability on utilitarian walking, with a positive association reported in two studies.	Based on data from four of the six studies, participants who lived in high walkable neighbourhoods compared to low walkable neighbourhoods accumulated 766 more steps per day.	MA was based on a relatively small number of studies. Associations for walkability was based on three large-scale features of neighbourhood designs (street connectivity, land use mix, residential density) however daily steps may be influenced by other components of walkability, for example, social cohesion.	Given the limited number of research, more studies using comparable exposure and outcome measurements are needed. The lack of comparable studies from adults in North America warrants further investigation as there is likely to be variations in the associations between neighbourhood walkability and levels of walking between continents.
**Hinkley T, 2008 (SLR)[48]**	Overall PA	Convenient play spaces; Availability of PA equipment/ toys/ play structures in school areas; Neighbourhood safety; Weather condition	To comprehensively investigate the correlates of preschool children's PA.	Only three physical environment variables were investigated in three or more studies, with largely inconclusive results found for outdoor condition (weather) on preschool children's PA. Time spent in play space and preschool	N.A.	A limited number of variables were investigated across 4 or more studies; limitations of included studies, for example, small, unrepresentative sample sizes limits generalisability of findings; methods used to assess PA may not be sensitive enough to detect associations in	Larger sample sizes may facilitate the identification of small, significant associations. It is key that future studies collect PA data across a range of contexts, times and locations. The use of validated measures of PA for the preschool population is important.

				attended were positively associated with PA.		this population, given the small sample sizes.	
**Koeneman MA, 2011 (SLR) [32]**	Overall PA	Access/ availability/ proximity recreational facilities; Access/ proximity parks/playgrounds /open space; Neighbourhood safety	To systematically review determinants of PA and exercise participation among healthy older adults.	Given the lack of high quality studies and that many studies only reported on one particular determinant, the review found insufficient evidence for the majority of associations.	N.A.	Combining studies that fell under the broad age range 'aged 55 and older' may have masked potential differences in determinants between subsamples; excluding subsamples based on disease or geographical location may have further masked important possible differences in determinants.	Important that valid, reliable measurement instruments are used to measure determinants and activity related outcome measures. Consistent methodology between studies will allow for comparisons of findings.
**Lachowycz K, 2011 (SLR) [33]**	Overall PA	Level of urbanization	To investigate the relationship between access to greenspace, obesity, and obesity-related health outcomes and behaviours.	Approximately two-thirds of studies reported a positive relationship, or some weak or mixed evidence of an association between greenspace and PA, with differences noted across different age ranges.	N.A.	Several papers reported data on same populations and were reported separately within this review which may lead to an overestimation of counts for particular findings.	Future research should focus on if and how people actually use greenspace. Investigating the mechanisms by which greenspace can improve health should be a research priority, in particular, if PA is one such mechanism. Developing a more solid socie-ecological framework would help researchers formulate and interpret research to date.

**Larouche R, 2014 (SLR) [40]**	Overall PA	Active school transportation	To examine differences in PA, body composition and cardiovascular fitness between active and passive travellers.	The majority of included studies showed positive associations between active school travel and PA levels, with moderate quality of evidence.	N.A.	Meta-analysis of studies was not possible due to the wide heterogeneity in study methodologies and analyses; inconsistencies in how active travel was defined and measured limit comparisons between studies.	Future studies should develop strategies to overcome the present limitations with methods used to measure active travel to school. More intervention studies are needed; the use of continuous measures of active travel will enable researchers to explore the dose response relationship between active travel and health related outcomes.
**Lee MC, 2008 (SLR) [37]**	Overall PA	Active means of transport to school	To explore the association between active commuting to school and outcomes of PA, weight and obesity in children.	12 studies reported a consistent positive association between active commuting and overall PA levels across many age groups of school children. The strength of the association was mixed across studies.	N.A.	Methods used within studies to classify children as 'active' or 'passive' commuters may have resulted in misclassification bias; majority of studies relied on self-reported measures of PA; conclusions based on cross-sectional data therefore causality cannot be established.	Future studies, in particular prospective quantitative analyses, are needed to determine if active commuting engenders PA in other aspects of children’s lives. Interventions that implement active commuting need to include measures of PA and other health related outcome measures to fully evaluate their efficacy.
**Maitland C, 2013 (SLR) [38]**	Overall PA	Access/Availability/Size of backyard space; Access/ Availability of play/ PA facilities and equipment in the home	To review the current evidence regarding the influence of the home physical environment on the sedentary behaviour and PA of children.	Only 6 studies of the physical environment included measures of the house and yard. PA equipment and the house and yard were not associated with PA, however environmental measures were exclusively self-reported within studies.	N.A.	Some outcome measures were pooled together making it difficult to draw conclusions on specific environmental measures; although study population was ‘pre-adolescent’, children and adolescents (as defined by previous reviews) were included which may limit comparability with	Future research should measure home context specific outcomes and how these interact with the social environment. Objective assessment of the physical environment at home is needed, including measures of the outdoor built environment.

						other reviews.	
**McCormack GR, 2011 (SLR) [31]**	Recreation walking, transportation walking, general walking, general cycling, combined walk/cycle, MVPA	Street connectivity; Presence of walking and cycling paths; Pedestrian/cycling amenities; Traffic related; Access/ proximity parks/playgrounds /open space; Access/ availability/ proximity recreational facilities; Non-recreational land use proximity; Availability/ Access/ Proximity of public transport system; Population/residential density; Land use mix; Environment aesthetics	To review quantitative studies examining associations between the built environment and PA.	Overall, neighbourhood design, population density, land use mix and connectivity were important determinants of PA. The built environment was more likely to be associated with transportation walking compared with other types of PA including recreational walking.	N.A.	Environmental attributes were grouped on face validity but some attributes may have been relevant to include under multiple groupings; methodological variations on how PA and the built environment were measured between studies makes it difficult to firmly conclude which attributes of the environment should be targeted in future interventions.	Future research should examine both the potential short and long-term effects of built environment interventions on PA related behaviours. Such interventions should also assess the cost-effectiveness of creating walkable neighbourhoods.

**McGrath LF, 2015 (MA) [39]**	MVPA	Active means of transport to school; Access/availability/proximity of destinations; Access/ proximity parks/playgrounds /open space; Street connectivity; Walkability; Availability of sidewalks/trails; Traffic Safety; Suburban vs urban	To conduct a SR and MA of studies linking aspects of the built environment with youth MVPA, including walking.	Neighbourhoods with features designed to promote PA had negative effects on children's MVPA whereas there were small-to-moderate positive effects on adolescents' PA. Urban space, for example, streets and hard surface play areas, were important for youth PA, with more activity occurring here compared with green spaces and parks.	Features of the built environment encouraging play were associated with trivial effects on 9-year-old children's activity (standardized effect -0.02; 90 % CI ±0.22), trivial–small effects on 12-year-olds' activity (0.17; ±0.10), and small effects on 15-year-old adolescent activity (0.35; ±0.17).	Results from the MA cannot quantify the additive effect when several features of the built-environment are provided in a given neighbourhood. Results the included studies were given the same weighting irrespective of demographics, days of recording, and proportion of days with useful data.	Given the limited evidence available on young children aged 5-8 years, future studies should include all stages of youth. Based on the negative associations observed for built environment features designed to promote PA in children, future research should examine whether parental restriction and lack of safe independent access to these are associated with lower levels of young children's PA.
**Olsen JM, 2013 (SLR) [29]**	Overall PA	Lack of access to PA equipment/facilities/programmes; Neighbourhood Safety; Lack of structures (sidewalks and streetlights)	To identify factors that influence PA levels in rural women.	Lack of access to facilities was noted as a barrier to PA, with rural women having less access than urban women. Rural women also experienced transportation difficulties when accessing PA	N.A.	Evaluation of data and analysis was done by one reviewer only; the terms 'rural' and 'PA' were inconsistently defined among studies; exclusion of articles studying women outside USA may mean some potentially relevant studies were	Interventions to promote PA in rural women should address the personal, socio-economic, and physical environment factors that influence PA. A consensus should be reached so that the terms 'rural' and 'physical activity' are consistently used across studies to strengthen knowledge in this area.

				facilities. Lack of safe places and lack of streetlights/sidewalks was a barrier to PA however 1 study found a negative association between sidewalks and PA.		excluded.	
**Pugliese J, 2007 (MA) [45]**	Overall PA	Access/Availability to family transport	To examine the direct effect of various parental behaviours on child and adolescent PA.	Instrumental behaviours, for example, transport, paying fees and purchasing equipment for PA were significantly related to child and adolescent PA, and do not appear to vary based on the age of participants.	N.A.	Variation between studies on how PA was defined/ measured may have influenced statistical analysis; ethnic and socioeconomic influences were not investigated.	Parental approaches to increasing PA in young children may become effective if they include parental modelling and encouragement of activity alongside instrumental influences. Future research, including longitudinal studies, should focus on more precise investigation of the contexts in which parental PA relates to children's PA.
**Rich C, 2012 (SLR) [36]**	Overall PA	Season/ Temperature	To undertake a review of published research evaluating the influence of season on accelerometer-determined sedentary behaviour and PA in children.	Seasonal variation in PA was reported in the majority of studies, particularly for children living in the UK, and in younger rather than older children. Findings from non-UK studies were inconsistent; seasonal variation in PA was reported in seven studies, but not in four and may be explained by differences in	N.A.	Limitations of included studies (small sample sizes, inconsistent study designs and accelerometer protocols, use of varied seasonal definitions) hampers conclusions; a number of included papers were drawn from the same cohort studies which may introduce bias; semi-quantitative nature of the review means conclusions cannot be drawn on strength of	Based on the findings of this review, there is sufficient evidence to develop public health interventions to increase PA during winter in UK children. Future research should be aimed at addressing the gaps in relation to seasonality and PA, in particular vigorous PA, and the factors that drive seasonal patterns, for example, age, sex, and geographic and climatic setting.

				climate.		the associations.	
**Ridgers ND, 2012 (SLR) [14]**	Recess PA	Access to outdoor space; Access to indoor activity space; Fixed equipment/markings; Unfixed equipment; Access/ provision of school facilities/ resources; Play location; Green spaces; Urban vs Rural school location; Season/temperature; Environment aesthetics	To examine correlates of children's and adolescents' PA during school recess periods.	Positive associations were found for overall facility provision and unfixed equipment, and recess PA. An indeterminate result was obtained for weather/seasonal differences, with no association found between available outdoor space and PA.	N.A.	Review limited by small sample size and cross-sectional nature of original studies; lack of consistency in measurement of PA therefore could not perform MA; lack of objective measures used to assess PA, particularly amongst adolescents.	The present review highlights a dearth of evidence in relation correlates of PA during school recess. Providing access to school facilities, providing unfixed equipment and identifying ways to promote encouragement for PA have the potential to inform strategies to increase PA levels during recess periods.
**Siddiqi Z, 2011 (SLR) [23]**	Overall PA	Availability/Access/Proximity of PA facilities/programmes; Access/ proximity parks/playgrounds /open space; Availability/ Access/ Proximity of public transport system; Lack of parks and open	To systematically examine and summarize factors impacting PA participation among African American adults.	Easy access to safe parks and recreational facilities were regarded as enablers to PA. Unsafe neighbourhoods were a consistent barrier amongst adults (18-50 years) and older adults (50+years).	N.A.	Review focused on African American adults therefore does not assess cross-cultural differences; only included qualitative studies therefore findings may not be generalizable to the wider population.	To effectively promote PA among African Americans, future targeted interventions need to address impediments at multiple levels, i.e. at the individual, social and environmental level. Furthermore, interventions should address the needs of subgroups within the African American community.

		space; Lack of access to PA equipment/facilities/programmes; Traffic density/ speed; Neighbourhood safety; Weather condition		Program availability at the community/church setting and easy access to open space and parks were facilitators among both age groups.			
**Stanley AM, 2012 (SLR) [35]**	School break time, after-school PA	Unfixed equipment, Fixed equipment/ markings, Size of play space; Access to play space; Green spaces; Access to areas that facilitate physical activity; Access to outdoor obstacle course; Access to indoor space; Access to a gym; Access/ provision of school facilities/ resources; Number of programs/activities; Access to seating; Design of the school grounds; Condition of field; Condition of facilities; Presence of lighting along	To identify the correlates of children's PA (aged 8–14 years) occurring during the school break time and after-school periods.	Access to a gym, access to four or more PA programs and the condition of a playing field were all associated with school break time PA in one study. Greater access to facilities were associated with higher levels of after-school PA in two or more studies	N.A.	A small number of studies with variation in important methodological aspects limits the generalisability of findings; cross-sectional nature of included studies means it is not possible to identify which correlates would provide the most powerful evidence for future intervention design.	Future research needs to focus on time-specific PA behaviour so that interventions designed for these specific periods can target the important correlates.

		paths; Presence of walking & cycling paths; Presence of other features (e.g. signage, trees); Park coverage; Availability of physical activity infrastructure/equipment; Neighbourhood safety; Environmental barriers to AT; Temperature = Season/ Temperature; Environment aesthetics; Land use mix diversity					
**Tzormpatzakis N, 2007 (SLR) [30]**	Overall PA, leisure time PA	Season/Temperature; Urban vs. rural residential location	To review the evidence from research relevant to participation in PA and exercise in Greece	In all European centres, total time dedicated to recreational activity in the summer was higher than in the winter. People living in urban areas were more likely to be sedentary compared to people living in rural areas.	N.A.	Measures of PA varied across studies and were not objective or validated for use in the Greek population; exercise and PA used interchangeably making associations between outcome measures and determinants difficult; most studies estimated seasonal participation based on a short data collection period (1-2 months), which may not be representative of PA over the whole	PA promotion should be organised in a systematic way; intervention studies and longitudinal designs to evaluate the long-term effects are suggested; a clear definition of variables is needed; studies should concentrate on the total PA profile of the participants.

						year.	
**Van der Horst K, 2007 (SLR) [49]**	Overall PA	Access/ Availability of play/ PA facilities and equipment in the home; Availability/Access/Proximity of PA equipment/facilities/programmes	To summarize and update the existing literature on correlates of young people's PA, insufficient PA, and sedentary behaviour	No significant associations were found between perceived access to facilities, perceived access to play space or for perceived access to sporting and/or fitness equipment at home and children's PA. No association between availability of facilities and adolescents PA was found.	N.A.	Main outcome measure was PA and the review did not differentiate between intensities of PA or PA at home versus school; owing to the cross-sectional nature and diversity of variables, samples, measures, and analyses, it was not possible to assess the overall strength of the associations.	More prospective studies are needed to gain greater insight into the correlates of change in PA levels. Majority of studies focused on adolescents therefore further research is needed on the determinants of PA in children.
**Van Holle V, 2012 (SLR) [24]**	Overall PA, leisure-time PA, total walking and/or cycling, recreational walking and/or cycling, active transportation in	Walkability; Pedestrian/ cycling amenities; Access/ availability/ proximity recreational facilities; Access/availability/proximity of destinations/ Availability/ Access/ Proximity of public transport system; Traffic	To summarise Europe-specific evidence on the relationship between the physical environment and different PA domains in adults	Convincing evidence on positive relationships with several PA domains was found for walkability, access to shops/services/work and environmental quality. Degree of urbanization showed contradictory results, dependent	N.A.	Could not calculate a number of PA-domain-specific summary results due to understudied factors; findings drawn from cross-sectional data therefore cannot ensure that convincing and possible evidence refers to causal relationships between the environment and PA. Majority of studies conducted in Western	More research assessing domain-specific relationships with several understudied environmental attributes (e.g., residential density) is needed. Studies should combine both objective and subjective environmental measures as these have been shown to relate differently to PA. More longitudinal studies in the research field are needed in order to reveal the influence of physical environmental attributes on different PA domains.

	general, transportation walking, transportation cycling	safety; Neighbourhood safety; Crime-related safety; Level of urbanization; Aesthetics; Quality of the environment; Land use mix diversity; Population/residential density		on the observed PA domain. Transportation PA was more frequently related to the physical environment than recreational PA.		Europe therefore caution should be paid when these generalising results to other geographical areas, such as Eastern Europe.	
**Wendel-Vos W, 2007 (SLR) [28]**	Overall PA, MVPA	Availability/Access/Proximity of PA facilities/programmes; Access/ availability/ proximity recreational facilities; Convenience of recreational facilities; Access/ Availability of PA infrastructure/ equipment; Availability of sidewalks/trails; Convenience of trails; Presence of streetlights; Satisfaction neighbourhood; Satisfaction neighbourhood services; Availability/	To gain insight into which environmental factors have been identified in observational studies as potential determinants of various types and intensity of PA among adult men and women.	Availability of PA equipment was convincingly associated with vigorous PA/ sports and connectivity of trails with active commuting. Other possible, but less consistent correlates of PA were availability, accessibility and convenience of recreational facilities. No evidence was found for differences between men and women.	N.A.	Most studies used cross-sectional designs and non-validated measures of environments and/or behaviour; majority of included studies based on data from the US/Australia therefore not clear whether or not associations found in these countries may be applicable to, for example, European countries.	Vast majority of environmental determinants included in this review resulted in null associations, implicating that there either is no association for these attributes or they were defined in a wrong way. Therefore, it is important to conduct future research with clear, possibly standardized definitions of environmental attributes and PA within the strongest study design possible.

		Access/ Proximity of public transport system; Traffic density/ speed; Traffic safety; Urban form; Land use mix diversity; Coastal location; Hills/steep road and terrain; Weather condition; Air/noise pollution; Environment score, Environment aesthetics					
