# Supplementary material for: A life course examination of the physical environmental determinants of physical activity behaviour: A “Determinants of Diet and Physical Activity” (DEDIPAC) umbrella systematic literature review
Source: PLoS One. 2017 Aug 7;12(8):e0182083. doi: 10.1371/journal.pone.0182083 (PMC5546676; doi:10.1371/journal.pone.0182083)
Supplement: S3 Table — (PDF) [file pone.0182083.s003.pdf]

S3 Table: Categorization of extracted potential physical environmental determinants

Table A: Categorization of extracted potential physical environmental determinants (preschool)

**MICRO-ENVIRONMENT**
**Home/Household**
**Access/Availability of outdoor toys/objects/equipment**
Outdoor: balls and objects (De Craemer)
Outdoor: open space (De Craemer)
Outdoor: Fixed equipment (De Craemer)
Outdoor: Wheel toys (De Craemer)
**Access/ Availability of play/ PA facilities and equipment in the home**
Availability of toys (De Craemer)
Play equipment at home (De Craemer)
**Access/Availability/Size of backyard space**
Backyard size (big) (De Craemer)
**Access/Availability to family transport (own more than one car)**
Own more than one car (De Craemer)
**Educational institutions**
**Distance to school**
Distance to school < 800m (De Craemer)
**Play space features**
Markings (De Craemer)
Vegetation (De Craemer)
Ground surface (De Craemer)
**Availability of PA equipment/ toys/ play structures in school areas**
Availability of toys (Hinkley)
Availability of toys (De Craemer)
Aiming equipment (De Craemer)
Playing equipment (De Craemer)
**Active means of transport to school (De Craemer)**
**Neighbourhood**
**Negative street characteristics**
Steep road on way to school (De Craemer)
No lights/crossings (De Craemer)
Busy road barrier on route to school (De Craemer)
**High traffic density/speed**
Heavy traffic (De Craemer)
**Availability/ Access/ Proximity of public transport system**
Public transport is limited (De Craemer)
**Presence of street lights**
Lighting along public open space paths (De Craemer)

**Access/ Availability of PA infrastructure/ equipment**
Equipment (De Craemer)
**Distance to PA facilities**
Miles to park (De Craemer)
**Access/ presence of parks/playgrounds/open space**
Convenient play spaces (Hinkley) (De Craemer)
Presence of public open spaces/playgrounds (De Craemer)
Number of recreational facilities in public open spaces (De Craemer)
**Neighbourhood Safety (Hinkley)**
**MACRO-ENVIRONMENT**
**City/Municipality/Regions**
**Environment aesthetics/ quality**
Environment (De Craemer)
**Season/ Temperature**
Season (De Craemer)
**Weather condition (favourable)**
Weather conditions (warmer/drier) (Hinkley)
**Preschool attended (rural/urban) (De Craemer)**

PA: Physical Activity; PE: Physical Education
Overall category in bold, additional variables contributing to category in italics

Table B: Categorization of extracted potential physical environmental determinants (children and adolescents)

**MICRO-ENVIRONMENT**
**Home/Household**
**Access/ Availability of play/ PA facilities and equipment in the home**
Home equipment (Davison)
PA equipment in home (Maitland)
Access/availability of exercise equipment (Ferreira)
Access/availability of PA equipment (Ferreira)
Access to facilities/ Home Equipment (Van der Horst)
**Access/Availability/Size of backyard space**
House and yard (Maitland)
**Access/Availability to family transport**
Own more than one car (De Craemer)
Instrumental parent support (transportation) (Beets)
Instrumental behaviors (transportation) (Pugliese)
Number of cars in household (Ferreira)
Transporting child (Gustafson)
**Educational institutions**
**Distance to school**
Distance to school (school location) (Davison)
Distance to school (Craggs)
Distance (from home) (Ferreira)
**Availability of PA equipment/ toys/ play structures in school areas**
Equipment/play structures in school play areas (Davison)
Availability of PA equipment (Ferreira)
Access to loose equipment (Stanley)
Unfixed equipment (Ridgers)
Access to fixed equipment (Stanley)
Fixed equipment/markings (Ridgers)
**Access/ provision of school facilities/ resources**
School facilities/ resources (Ferreira)
Overall facility provision (Ridgers)
Access to facilities (Stanley)
Number of facilities (Stanley)
**Number of PA programs/ activities (Stanley)**
**Access to seating (Stanley)**
**Access to play space**
Access to play space (Stanley)
Indoor space/areas (Ridgers)
Access to indoor activity space (Stanley)
Access to outdoor space (Ridgers)
Outdoor physical activity areas (Ridgers)

Outdoor space (Ridgers)
Play location (indoor) (Ridgers)
**Access to areas that facilitate PA**
Access to court space (Stanley)
Access to playing fields (with markings) (Stanley)
Access to sledding hill (Stanley)
Access to ski tracks (Stanley)
Access to ice-skating areas (Stanley)
Access to fenced courtyard space (Stanley)
Access to climbing wall (Stanley)
Access to a wooded area (Stanley)
Access to water (sea, river, lake) (Stanley)
Access to bitumen areas (Stanley)
Access to areas for hopscotch/skipping (Stanley)
Access to areas for board/skating (Stanley)
Access to swimming facilities (Stanley)
**Access to outdoor obstacle course (Stanley)**
**Play space features**
Access to green space (no markings) (Stanley)
Play surface (grass) (Ridgers)
Playground markings (Stanley)
Size of play space (Stanley)
Design of the school grounds (Stanley)
**Access to a gym with cardio & weightlifting equipment (Stanley)**
**Condition of facilities**
Condition of facilities (Stanley)
Condition of a gymnasium (Stanley)
Condition of field (Stanley)
**Active means of transport to school**
Walking to school (McGrath)
Active commuting to school (Lee)
Active school transportation (Larouche)
**Environmental barriers to active travel (Stanley)**
**Neighbourhood**
**Neighbourhood Design**
Range of Housing Opportunities and Choices (Durand)
**Access/distance/proximity to destinations**
Access to destinations (Davison)
Proximity of neighhourhood shops (McGrath)
Distance to destinations (Ferreira)
**Street characteristics**
Street characteristics (Davison)
Road characteristics (Craggs)

**Street length**
Road length (Craggs)
**Negative street characteristics**
Steep terrain (Davison)
**Availability of sidewalks/trails**
Presence of sidewalks (Davison)
Provision of sidewalks (McGrath)
**Street Connectivity**
Connectivity of street network (Davison)
Number of sidewalks (McGrath)
Street connectivity (D'Haese) (Ding)
**Footpath conditions/ available shelters**
Street and sidewalk conditions (Davison)
Available shelters/foot path conditions (Ferreira)
**Presence of Walking and Cycling Paths/Amenities**
Presence of bike lanes/ease of cycling (Davison)
Presence of walking & cycling paths (Stanley)
Walk/ cycle facilities (D'Haese) (Ding)
**Pedestrian and cyclist safety structure**
Pedestrian and cyclist safety (Davison)
Pedestrian safety structures (Ding)
Presence of controlled crossings (Davison)
**Number of roads to cross**
Number of roads to cross (Davison)
**Traffic density/speed**
Traffic speed/volume (Ding)
Traffic (density/speed) (Davison)
Road traffic (Craggs)
**Walkability**
Walkability (McGrath) (D'Haese) (Ding)
Walkable neighbourhoods (Durand)
**Accessiblity (D'Haese)**
**Traffic safety (D'Haese)**
**Traffic related hazards**
Traffic infrastructure (McGrath)
Neighbourhood hazards (e.g. many roads/no lights crossings; heavy traffic; physical disorder; pollution) (Ferreira)
**Availability/ Access/ Proximity of public transport system**
Availability of public transportation (Davison)
Limited public transport (Ferreira)
Variety of Transportation Choices (Durand)
**Presence of street lights**
Presence of lighting along paths (Stanley)
**Availability of physical activity infrastructure/equipment**
Availability of physical activity infrastructure/equipment (Craggs)

Access to equipment (Stanley)
**Availability/Access/Proximity of PA facilities/programmes**
Access to facilities (Stanley)
Access/availability to PA facilities/programmes (Ferreira)
Access/availability to PA equipment/facilities/programmes (Ferreira)
Availability of facilities(Van der Horst)
Number of amenities (Stanley)
**Access/ availability/ proximity recreational facilities**
Availability recreation facilities (Davison)
Recreation faciliites (D'Haese)
Recreation facilities (access/density/proximity) (Ding)
**Distance to PA facilities**
Distance to PA facilities (Ferreira)
**Access/ proximity parks/playgrounds/open space**
Proximity of playgrounds and parks (Davison)
Neighbourhood play space (McGrath)
Parks (access/density/proximity) (Ding)
Open Space and Critical Environmental Areas (Durand)
Park coverage (Stanley)
**Presence of other features (e.g. signage, trees)**
Presence of trees (Stanley)
Presence of shade (Stanley)
Presence of a water feature (Stanley)
Presence of signage re dogs (Stanley)
Presence of signage restricting other activities (Stanley)
**Neighbourhood Safety**
Perceived safety (Davison)
Neighbourhood Safety (Craggs) (Stanley)
Safety (D'Haese)
**Crime safety (D'Haese)**
**Neighbourhood physical disorder**
Physical disorder/tidiness of area (Davison)
Neighbourhood physical disorder (Ferreira)
**MACRO-ENVIRONMENT**
**City/Municipality/Regions**
**Season/ Temperature**
Month of year (average temperature) (Davison)
Seasonal variation (Rich)
Seasons/temperature/weather (Ridgers)
Temperature (Stanley)
Season (spring, summer) (Ferreira)
**Weather condition (unfavourable)**
Unsuitable weather (Davison) (Ferreira)

**Land use mix diversity**
Land use mix (Stanley) (Ding)
Mix land uses (Durand)
Land use mix diversity (D'Haese)
**Population/ residential density**
Population/ residential density (Davison)
Density (D'Haese)
Residential density (Ding)
**Urban Form**
Compact buidling design (Durand)
**Urban vs Rural residential location**
Rural/suburban versus urban (Davison)
Urban vs. rural (Ferreira)
**Urban vs suburban (McGrath) (Ferreira)**
**Rural school location**
School location (rural) (Ridgers)
**Environment aesthetics/quality**
Aesthetics of neighborhood (Davison)
Aesthetics in the environment (Craggs)
Aesthetics (Ridgers) (Stanley) (D'Haese)
**Vegetation (presence of street trees) (Ding)**
**Level of urbanization**
Level of urbanization (Lachowycz) (Ferreira)
**Coastal location**
Coastal vs. mountains (Ferreira)

PA: Physical Activity; PE: Physical Education
Overall category in bold, additional variables contributing to category in italics

Table C: Categorization of extracted potential physical environmental determinants (adults)

**MICRO-ENVIRONMENT**
**Neighbourhood**
**Neighbourhood Design**
Range of Housing Opportunities and Choices (Durand)
**Access/availability/proximity of destinations**
Access to shops/services/work (Van Holle)
**Negative street characteristics**
Lack of sidewalks and streetlights (Olsen)
Hills (Wendel-Vos)
Hilliness (Van Holle)
**Street Connectivity**
Street/pedestrian connectivity (McCormack)
**Availability/Presence of Walking and Cycling Paths/Amenities**
Trails/pathways/cycle ways/sidewalk (McCormack)
Walking/cycling facilities (Van Holle)
Pedestrian/cyclist amenities (street furniture, lighting, shading) (McCormack)
Availability of sidewalks (Wendel-Vos)
Availability of trails (Wendel-Vos)
Presence of sidewalks (Casagrande)
Convenience of trails (Wendel-Vos)
**Traffic density/speed**
Traffic volume (Wendel-Vos)
Light traffic (Casagrande)
Heavy traffic (Siddiqi)
**Walkability**
Walkability (Van Holle) (Hajna)
Walkable neighbourhoods (Durand)
**Traffic safety**
Traffic-related safety (Van Holle)
Traffic safety (Wendel-Vos)
Traffic related (McCormack)
**Availability/ Access/ Proximity of public transport system**
Access to public transport (Van Holle)
Convenience of public transport (Wendel-Vos)
Variety of Transportation Choices (Durand)
Lack of public transportation (Siddiqi)
Transit proximity/access (McCormack)
**Presence of streetlights**
Streetlights (Wendel-Vos)
**Availability/Access/Proximity of PA facilities/programmes/equipment**
Availability of PA equipment (Wendel-Vos)
Accessibility of facilities (Wendel-Vos)
Places to exercise (Casagrande)

Program availability at church/community (Siddiqi)
**Access/ availability/ proximity recreational facilities**
Access to recreational Facilities (Van Holle)
Availability of recreational facilities (Wendel-Vos)
Accessibility of recreational facilities (Wendel-Vos)
Recreation land use proximity (McCormack)
Convenience recreational facilities (Wendel-Vos)
**Non-recreational land use proximity (McCormack)**
**Access/ proximity parks/playgrounds/open space**
Access to open space (Babacus)
Easy access to parks (Siddiqi)
Parks/public open space install or improvements (McCormack)
**Lack of access to PA equipment/facilities/programmes**
Lack of Access (Transportation difficulties,lack of resources, lack of affordability) (Olsen)
Culturally inappropriate facilities (Babacus)
Lack of facilities (Siddiqi)
**Lack of parks and open space (Siddiqi)**
Open Space and Critical Environmental Areas (Durand)
**Neighbourhood Satisfaction**
Satisfaction neighbourhood (Wendel-Vos)
Satisfaction neighbourhood services (Wendel-Vos)
**Neighbourhood Safety**
Lack of Safety (lack a safe place: busy road, weather (ice & heat), dogs and wild animals) (Olsen)
Safety (Van Holle)
Unsafe neighbourhood (Siddiqi)
Neighbourhood safety for walking (Koeneman)
**Safety from crime (Van Holle)**
**Environmental barriers of PA (Casagrande)**
**MACRO-ENVIRONMENT**
**City/Municipality/Regions**
**Weather condition (unfavourable)**
Poor weather (Babacus)
Bad weather (Wendel-Vos)
Adverse weather/climate conditions (Siddiqi)
**Season/ Temperature**
Season (Tzormpatkis)
**Land use mix diversity**
Land use mix (Wendel-Vos) (McCormack)
Mix land uses (Durand)
Land use mix diversity (Van Holle)
**Population/ residential density**
Residential density (Van Holle)
Population/residential density (McCormack)

**Urban Form**
Compact buidling design (Durand)
Urban sprawl (Wendel-Vos)
Urban form (Casagrande)
**Urban vs Rural residential location**
Living in rural/ urban area (Tzormpatkis)
**Environment aesthetics/quality**
Aesthetics (Van Holle)
Environment aesthetics (Wendel-Vos)
Physical environment (Coble)
Aesthetics/variety/diversity (McCormack)
**Quality of the environment (Van Holle)**
**Environment score (Wendel-Vos)**
**Air/noise pollution (Wendel-Vos)**
**Level of urbanization**
Level of urbanization (Lachowycz)
Urbanization (Van Holle)
**Coastal location**
Coastal location (Wendel-Vos)
